# Association between food intake and obesity in pregnant women living with and without HIV in Cape Town, South Africa: a prospective cohort study

**DOI:** 10.1186/s12889-021-11566-2

**Published:** 2021-08-04

**Authors:** Hlengiwe P. Madlala, Nelia P. Steyn, Emma Kalk, Mary-Anne Davies, Dorothy Nyemba, Thokozile R. Malaba, Ushma Mehta, Gregory Petro, Andrew Boulle, Landon Myer

**Affiliations:** 1 Division of Epidemiology and Biostatistics, School of Public Health and Family Medicine, University of Cape Town, Falmouth Building, Anzio Road, Observatory, Cape Town, Western Cape 7925 South Africa; 2 Division of Human Nutrition, Department of Human Biology, University of Cape Town, Cape Town, Western Cape South Africa; 3 Centre for Infectious Disease Epidemiology and Research, School of Public Health and Family Medicine, University of Cape Town, Cape Town, Western Cape South Africa; 4 Health Impact Assessment Unit, Western Cape Department of Health, Cape Town, South Africa; 5 Department of Obstetrics and Gynaecology, University of Cape Town and New Somerset Hospital, Cape Town, Western Cape South Africa

**Keywords:** Food intake, Body mass index, Gestational weight gain, Pregnancy, HIV

## Abstract

**Background:**

Although global nutrition/dietary transition resulting from industrialisation and urbanisation has been identified as a major contributor to widespread trends of obesity, there is limited data in pregnant women, including those living with HIV in South Africa. We examined food-based dietary intake in pregnant women with and without HIV at first antenatal care (ANC) visit, and associations with maternal overweight/obesity and gestational weight gain (GWG).

**Methods:**

In an urban South African community, consecutive women living with (*n* = 479) and without (*n* = 510) HIV were enrolled and prospectively followed to delivery. Interviewer-administered non-quantitative food frequency questionnaire was used to assess dietary intake (starch, protein, dairy, fruits, vegetables, legumes, oils/fats) at enrolment. Associations with maternal body mass index (BMI) and GWG were examined using logistic regression models.

**Results:**

Among women (median age 29 years, IQR 25–34), the prevalence of obesity (BMI ≥ 30 kg/m^2^) at first ANC was 43% and that of excessive GWG (per IOM guidelines) was 37% overall; HIV prevalence was 48%. In women without HIV, consumption of potato (any preparation) (aOR 1.98, 95% CI 1.02–3.84) and pumpkin/butternut (aOR 2.13, 95% CI 1.29–3.49) for 1–3 days a week increased the odds of overweight/obesity compared to not consuming any; milk in tea/coffee (aOR 6.04, 95% CI 1.37–26.50) increased the odds of excessive GWG. Consumption of eggs (any) (aOR 0.52, 95% CI 0.32–0.86) for 1–3 days a week reduced the odds of overweight/obesity while peanut and nuts consumption for 4–7 days a week reduced the odds (aOR 0.34, 95% CI 0.14–0.80) of excessive GWG.

In women with HIV, consumption of milk/yoghurt/maas to drink/on cereals (aOR 0.35, 95% CI 0.18–0.68), tomato (raw/cooked) (aOR 0.50, 95% CI 0.30–0.84), green beans (aOR 0.41, 95% CI 0.20–0.86), mixed vegetables (aOR 0.49, 95% CI 0.29–0.84) and legumes e.g. baked beans, lentils (aOR 0.50, 95% CI 0.28–0.86) for 4–7 days a week reduced the odds of overweight/obesity; tomato (raw/cooked) (aOR 0.48, 95% CI 0.24–0.96) and mixed vegetables (aOR 0.38, 95% CI 0.18–0.78) also reduced the odds of excessive GWG.

**Conclusions:**

Diet modification may promote healthy weight in pregnant women living with and without HIV.

**Supplementary Information:**

The online version contains supplementary material available at 10.1186/s12889-021-11566-2.

## Introduction

One of the targets for Sustainable Development Goal (SDG) 3 is ‘reducing by one third premature mortality from non-communicable diseases (NCDs) through prevention and treatment’. In South Africa, almost 70% of women over 15 years are overweight/obese, the highest prevalence in sub-Saharan Africa (SSA) [1]. Consequently, over 40% of pregnant women are obese at antenatal care (ANC) entry, including those living with human immunodeficiency virus (HIV) on lifelong antiretroviral therapy (ART) [2–5]. Abundant data show that maternal obesity is associated with metabolic complications in pregnancy, which later increase the risk of a range of NCDs during postpartum period [6–8]. Further, maternal obesity is associated with adverse birth outcomes and is implicated in foetal ‘metabolic programming’, which over time may be associated with metabolic complications in offspring [9–11]. Therefore, efforts directed at reducing obesity in pregnancy may have a significant impact in achieving some targets of SDG 3 by 2030.

Global nutrition/dietary transition as a result of industrialisation and urbanisation has been identified as a major contributor to widespread trends of obesity [12]. Specifically, consumption of high calorie-rich foods which have poor micronutrient content has been shown to be associated with overweight/obesity [13]. In an urban setting in South Africa, mapping of household food environments revealed a large proportion (71%) of households that frequently consume obesity-risky foods such as bread and processed meat, and low consumption (16%) of protective foods such as fruits and vegetables [14]. Although several studies have reported dietary intake in South Africa [15–17], there is limited data in pregnant women, including those living with HIV.

During pregnancy, there is increased demand for nutrient supply to support the growth and development of the foetus, thus dietary intake of pregnant women may be different from the general population. Additionally, African women have a cultural belief of needing to ‘eat for two’ during pregnancy, which may increase the risk of excessive gestational weight gain (GWG) and subsequent postpartum NCDs in mothers and their children [18]. Therefore, research on dietary food intake in pregnant women is critical for the mission of curbing NCDs, particularly because diet is a modifiable lifestyle factor. The objective of the present study was to examine food-based dietary intake in pregnant women with and without HIV at first ANC visit in Cape Town, and associations with maternal obesity and GWG.

## Methods

Consecutive pregnant women (≥18 years) attending first ANC services at a large primary health care facility, Gugulethu Community Health Centre, were enrolled into a prospective cohort (*n* = 989, with HIV [*n* = 479] and without HIV [*n* = 510]) between January 2017 and July 2018 and followed through to delivery (February 2019). For the study, participants were actively followed through face-to-face interviews with intensive measurements that took place during the first, second and third trimesters of pregnancy.

A 30-item non-quantitative Food Frequency Questionnaire (FFQ) was employed to assess dietary intake in the past 7 days prior to first ANC visit. To minimise burden on study participants, a 61-item FFQ designed for use in South African context [19, 20] was adapted to 30-items assessed in this study. The limited food items included were selected because they are amongst the most commonly consumed in each of the food groups examined in the parent questionnaire. Although the parent FFQ has not been previously used in pregnant women, we have no reason to believe that the food items assessed necessarily change substantially in pregnancy other than the portion sizes which were not investigated in this study. The 30 food items assessed included 7 food groups, namely starch (brown/whole wheat bread/rolls, breakfast cereal [instant], oats porridge, sweet potato and potato [any preparation]), protein (red meat [any], organ meat e.g. liver, chicken [any], tinned fish and eggs [any]), dairy (milk/yoghurt/maas to drink/on cereals, milk in tea/coffee and cheese [except cottage]), fruits (citrus fruit e.g. orange, pure orange/guava juice, banana, mangoes, apples/pears and avocado), vegetables (broccoli, spinach [including morogo], carrots, tomato [raw/cooked], green beans, green peas, mixed vegetables, pumpkin/butternut), legumes (legumes e.g. baked beans, lentils and peanut and nuts) and fats/oils (soft margarine [tub]). There were four responses to frequency of consumption – ‘never’, ‘1–3 days’, ‘4–6 days’ and ‘7 days’. In analysis, category ‘7 days’ was combined with ‘4–6 days’ resulting in three categories with a third category named ‘4–7 days’.

Alcohol use in pregnancy was defined as alcohol consumption after finding out about the pregnancy. Socioeconomic status (SES) was represented as a composite score based on education level, employment status, type of housing, and presence of a toilet, running water, electricity, fridge, telephone and television in the house [21]; the scores were categorised into tertiles corresponding to lowest, middle and highest SES group. The study protocol was reviewed and approved by the Faculty of Health Sciences Human Research Ethics Committee of the University of Cape Town (REF 541/2015) and the Western Cape Department of Health (REF WC_2016RP6_286). Written informed consent was obtained from all participants at enrolment and all procedures complied with the Helsinki Declaration as revised in 1983.

### Outcome assessment

Outcomes of the study were maternal BMI and GWG. At first ANC, which coincided with enrolment, weight and height measurements were performed by a trained study nurse using a calibrated scale (Charder, Taichung City, Taiwan) accurate to within 0.5 kg; height measurements were taken to the nearest 0.1 cm using a stadiometer (Seca, Birmingham, United Kingdom). Using a method described by Santos et al. [22], a correction factor on weight measured at first ANC visit was applied based on gestational age (GA) at measurement. Briefly, using international standards for GWG in pregnancy [22], the median weight gained for each week of gestation was subtracted from the weight measured at first ANC visit based on BMI category. Using this corrected weight, the estimated BMI was then calculated as weight divided by squared height; and categorised as underweight (< 18.5), normal (18.5–24.9), overweight (25–29.9) and obese (≥30) in kg/m^2^). Due to small percentage of underweight women, this category was combined with normal BMI in the regression. Further, in the regression, overweight and obese categories were combined for simplicity. For GWG, second and third trimester weight measurements were taken subsequent to the enrolment weight. Weekly maternal GWG was calculated by dividing the weight change between enrolment and second or third study visit by the number of weeks elapsed between the two intervals [23], and expressed as kg/week. Weekly GWG was categorised as inadequate, adequate and excessive based on the Institute of Medicine (IOM)-recommended GWG ranges which vary by BMI category [24].

### Statistical analysis

Data were analysed using STATA version 15.0 (Stata Corporation, College Station, TX, USA). Maternal baseline characteristics and frequency of food-based dietary consumption were stratified by HIV status, BMI and GWG categories. Differences between groups were compared using Chi-Squared test. Associations between the frequency of consumption of the 30 individual food items and maternal BMI as well as GWG in women with and without HIV were examined using multivariable logistic regression models. Results are presented as adjusted odds ratios (aOR) with related 95% confidence intervals (CI). Adjusted models included a priori confounders such as age, SES, relationship status, alcohol use and parity.

**Fig. 1:**
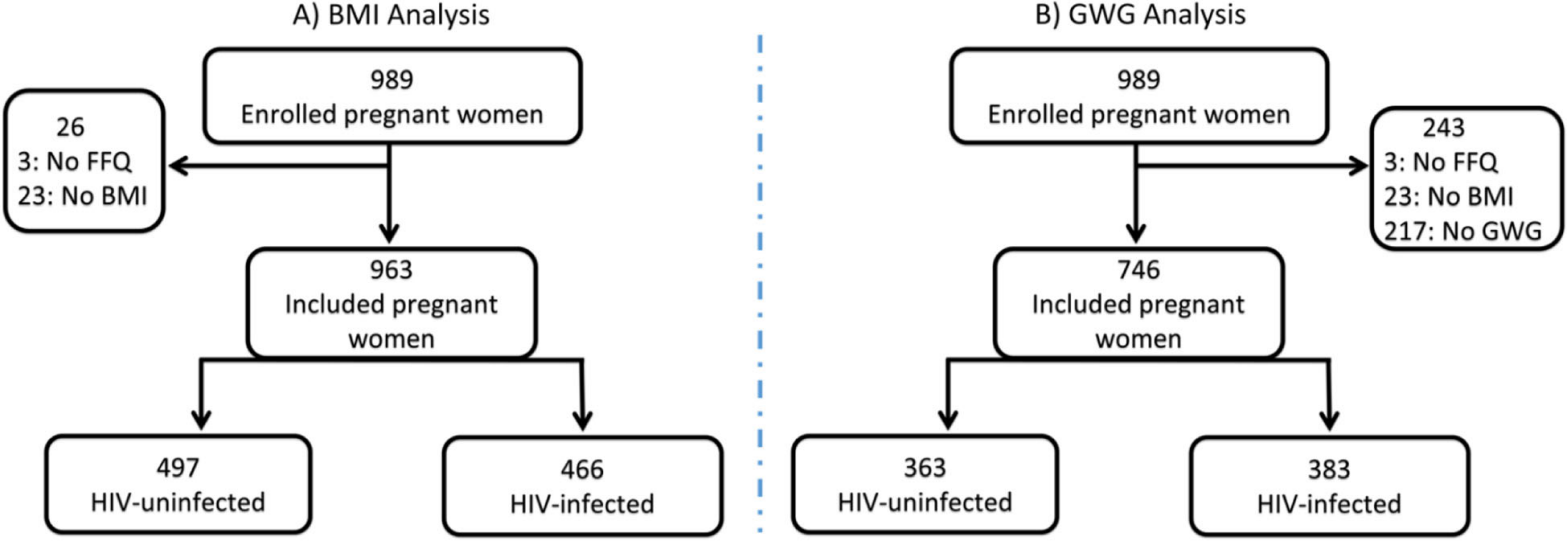
Flow diagram showing the selection of women with and without HIV included in the BMI (**A**) and GWG (**B**) regression analysis

Regression analyses included women with complete data on exposure and outcomes of interest i.e. food consumption frequency, BMI (*n* = 963) and GWG (*n* = 746). Participants with missing data on food consumption frequency (*n* = 3; 0.3%), weight/height/GA for BMI calculation (*n* = 23; 2%) and second or third visit weight for GWG calculation (*n* = 217; 22%) were excluded from the regression (Fig. 1). For confounders, missing data were included in the reference category as appropriate.

## Results

The mean age of 989 participants enrolled was 29 years (SD ±6), gestational age at first ANC visit was 20 weeks (IQR, 14–25), 25% of women were primigravid and 48% were living with HIV. The median BMI was 29 kg/m^2^ (IQR, 24–34) overall; there were 21 (2%) women with underweight, 273 (28%) normal weight, 244 (25%) overweight and 427 (43%) obese BMI. Of the 427 women with obesity, 236 (46%) were living without HIV and 191 (40%) were living with HIV (Additional file 1). The median GWG was 0.36 kg/week (IQR, 0.14–0.54) overall, there were 266 (27%) women with inadequate, 121 (12%) adequate and 362 (37%) excessive GWG. Of the 362 women with excessive GWG, 199 (39%) were living without HIV and 163 (34%) were living with HIV (Additional file 1).

**Fig. 2:**
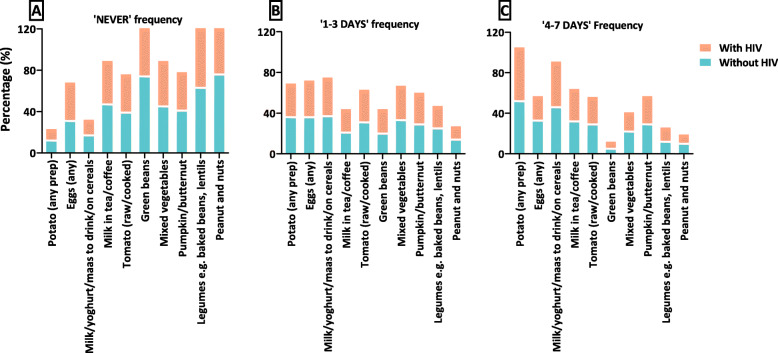
Consumption frequencies of selected food items in women living with and without HIV for categories ‘never’ (**A**), ‘1–3 days’ (**B**) and ‘4–7 days’ (**C**) in the past week

Table 1 describes maternal baseline characteristics overall and stratified by HIV status, BMI and GWG categories. Women with HIV were more likely to be older and had higher parity compared to women without HIV, as were women with obesity compared to women with normal BMI. Women with excessive GWG were less likely to present early for their first ANC. Additional file 2 shows consumption frequencies of 30 food items overall and stratified by HIV status, BMI and GWG categories; Fig. 2 shows selected food items stratified by HIV status only. The most frequently consumed (‘4–7 days’) starch was brown/whole wheat bread/rolls (58%) and potatoes (any preparation) (53%). Women with excessive GWG were more likely to consume potatoes for 4–7 days compared to those with adequate GWG (60% vs 47%, *p* = 0.045). Of note, sweet potatoes (8%) were the least consumed starch; women with obesity were more likely to report no consumption of sweet potatoes compared to women with normal BMI (84% vs 76%, *p* = 0.013). The most frequently consumed protein was chicken (48%) and eggs (29%); women with HIV were less likely to consume eggs for 4–7 days compared to women without HIV (25% vs 33%, *p* = 0.007) (Fig. 2). Organ meat e.g. liver (13%) and tinned fish (15%) were the least consumed proteins. The most frequently consumed vegetable was carrots (35%), with broccoli being the least (5%). Women with obesity were more likely to report no consumption of broccoli (90% vs 82%, *p* = 0.019), but more likely to consume pumpkin/butternut (32% vs 26%, *p* = 0.028) for 4–7 days compared to women with normal BMI. Women with HIV were less likely to report no consumption of spinach (47% vs 54%, *p* = 0.047) and green beans (67% vs 74%, *p* = 0.025) compared to women without HIV (Fig. 2). However, women with excessive GWG were more likely to report no consumption of spinach compared to those with adequate weight gain (56% vs 48%, *p* = 0.026). Both legume items (baked beans/lentils [14%]; peanuts/nuts [10%]) were least consumed and this was similar by HIV, BMI and GWG categories.

**Table 1:** Maternal baseline characteristics of women included in the analysis, overall and stratified by HIV status, BMI and GWG category

		HIV status			BMI				GWG			
Characteristic	Overall N (%) = 989	Without HIV N (%) = 510	With HIV N (%) = 479	*p* value	Normal N (%) = 294	Overweight N (%) = 244	Obese N (%) = 427	*p* value	Inadequate N (%) = 266	Adequate N (%) = 121	Excessive N (%) = 362	*p*-value
Age (years)				**< 0.001**				**< 0.001**				0.077
< 24	240 (24)	171 (34)	69 (14)		100 (34)	58 (24)	79 (19)		48 (18)	30 (25)	100 (28)	
25–29	292 (30)	157 (31)	135 (28)		93 (32)	71 (29)	120 (28)		93 (35)	35 (29)	104 (29)	
30–34	254 (26)	109 (21)	145 (30)		71 (24)	54 (22)	123 (29)		75 (28)	34 (28)	81 (22)	
≥ 35	203 (21)	73 (14)	130 (27)		30 (10)	61 (25)	105 (25)		48 (18)	22 (18)	76 (21)	
Median (IQR)	29 (25–34)	27 (23–32)	31 (26–35)		27 (23–32)	29 (25–35)	30 (26–34)		29 (26–33)	29 (25–33)	29 (24–33)	
Education				**0.015**				0.870				0.661
Primary	37 (4)	15 (3)	22 (5)		9 (3)	10 (4)	17 (4)		9 (3)	4 (3)	17 (5)	
High school	929 (94)	477 (94)	452 (94)		277 (94)	231 (95)	399 (93)		252 (95)	113 (93)	334 (93)	
Tertiary	23 (2)	18 (4)	5 (1)		8 (3)	3 (1)	11 (3)		3 (1)	4 (3)	10 (3)	
Socio-economic status				0.071				0.602				0.646
Lower	325 (33)	152 (30)	173 (36)		105 (36)	72 (30)	136 (32)		87 (33)	45 (37)	119 (33)	
Middle	274 (28)	142 (28)	132 (28)		75 (26)	71 (29)	123 (29)		81 (31)	35 (29)	96 (27)	
Higher	388 (39)	215 (42)	173 (36)		113 (38)	100 (41)	168 (39)		96 (36)	41 (34)	144 (40)	
Missing	2 (0.2)	1 (0.2)	1 (0.2)		1 (0.3)	1 (0.4)	0		0	0	2 (0.6)	
Relationship status				0.874				**0.001**				0.271
No relationship	46 (5)	23 (5)	23 (5)		13 (4)	11 (5)	22 (5)		9 (3)	1 (1)	17 (5)	
Not Cohabiting/married-NLT	509 (51)	267 (52)	242 (51)		180 (61)	124 (51)	194 (45)		140 (53)	72 (60)	186 (52)	
Cohabiting/married-LT	428 (43)	218 (43)	210 (44)		98 (33)	106 (43)	211 (49)		114 (43)	48 (400	155 (43)	
Missing	6 (1)	2 (0.4)	4 (1)		3 (1)	3 (1)	0		2 (1)	0	3 (1)	
*Alcohol use				0.982				0.599				0.227
No	898 (91)	463 (91)	435 (91)		264 (90)	218 (89)	392 (92)		243 (92)	114 (94)	322 (89)	
Yes	89 (9)	46 (9)	43 (9)		29 (10)	25 (10)	35 (8)		21 (8)	7 (6)	38 (11)	
Missing	2 (0.2)	1 (0.2)	1 (0.2)		1 (0.3)	1 (0.4)	0		1 (0.4)	0	1 (0.3)	
GA at first ANC (weeks)				0.109				0.455				**0.002**
1st trimester (≤13)	229 (23)	109 (21)	120 (25)		64 (22)	54 (22)	110 (26)		74 (28)	39 (32)	64 (18)	
2nd trimester (14–28)	609 (62)	314 (62)	295 (62)		183 (62)	160 (66)	265 (62)		162 (61)	73 (60)	244 (68)	
3rd trimester (> 28)	123 (12)	73 (14)	50 (10)		44 (15)	29 (12)	49 (11)		28 (11)	8 (7)	51 (14)	
Missing	28 (3)	14 (3)	14 (3)		3 (1)	1 (0.4)	3 (0.7)		2 (1)	1 (1)	3 (1)	
Median (IQR)	20 (14–25)	21 (15–26)	19 (13–24)		21 (14–26)	20 (14–23)	19 (13–24)		19 (13–24)	18 (12–22)	21 (15–25)	
Parity				**< 0.001**				**< 0.001**				0.690
0	251 (25)	168 (33)	83 (17)		106 (36)	51 (21)	86 (20)		57 (22)	33 (27)	94 (26)	
1	331 (33)	169 (33)	162 (34)		92 (31)	89 (36)	143 (33)		93 (35)	40 (33)	117 (32)	
≥ 2	407 (41)	173 (34)	234 (49)		96 (33)	104 (43)	198 (46)		114 (43)	48 (40)	150 (42)	
Median (IQR)	1 (0–2)	1 (0–2)	1 (1–2)		1 (0–2)	1 (1–2)	1 (1–2)		1 (1–2)	1 (0–2)	1 (0–2)	

* In current pregnancy. Married-NLT – Married but not living together, Married-LT – married and living together, GA – gestational age, ANC – antenatal care. BMI – body mass index, GWG – gestational weight gain. Missing BMI 23 (2.4%), GWG 240 (24.3%)

**Fig. 3:**
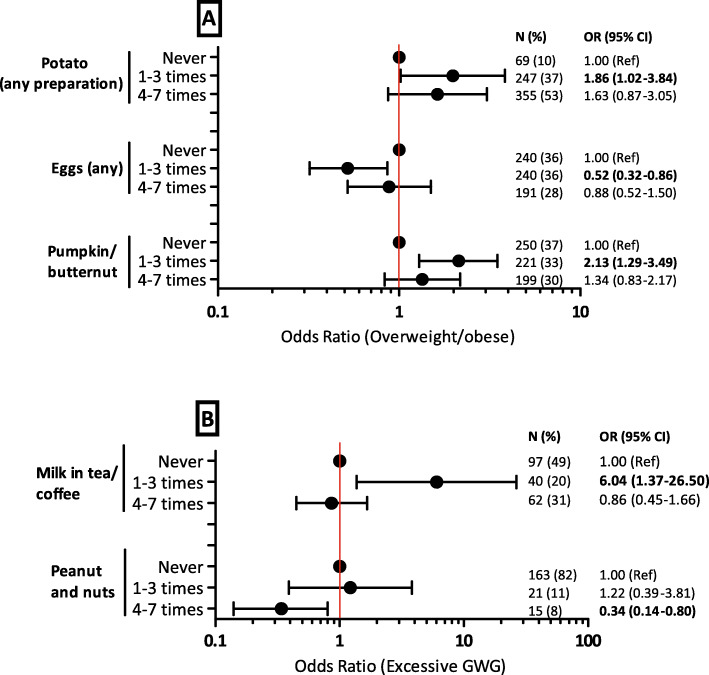
Associations between food consumption frequency and overweight/obesity (**A**) and excessive GWG (**B**) in women without HIV

Figure 3 shows the association between food intake frequency of selected items and overweight/obesity (A) and excessive GWG (B) in women without HIV (30 food items shown in Additional files 3 and 4). Consumption of potato (any preparation) (aOR 1.98, 95% CI 1.02–3.84) and pumpkin/butternut (aOR 2.13, 95% CI 1.29–3.49) for 1–3 days a week was positively associated with increased odds of overweight/obesity compared to not consuming any in the past week. Consumption of milk in tea/coffee (aOR 6.04, 95% CI 1.37–26.50) for the same frequency was positively associated with increased odds of excessive GWG. In contrast, consumption of eggs (any) (aOR 0.52, 95% CI 0.32–0.86) for 1–3 days a week was negatively associated with reduced odds of overweight/obesity, while peanut and nuts consumption for 4–7 days a week was negatively associated with reduced odds (aOR 0.34, 95% CI 0.14–0.80) of excessive GWG compared to not consuming any in the past week.

**Fig. 4:**
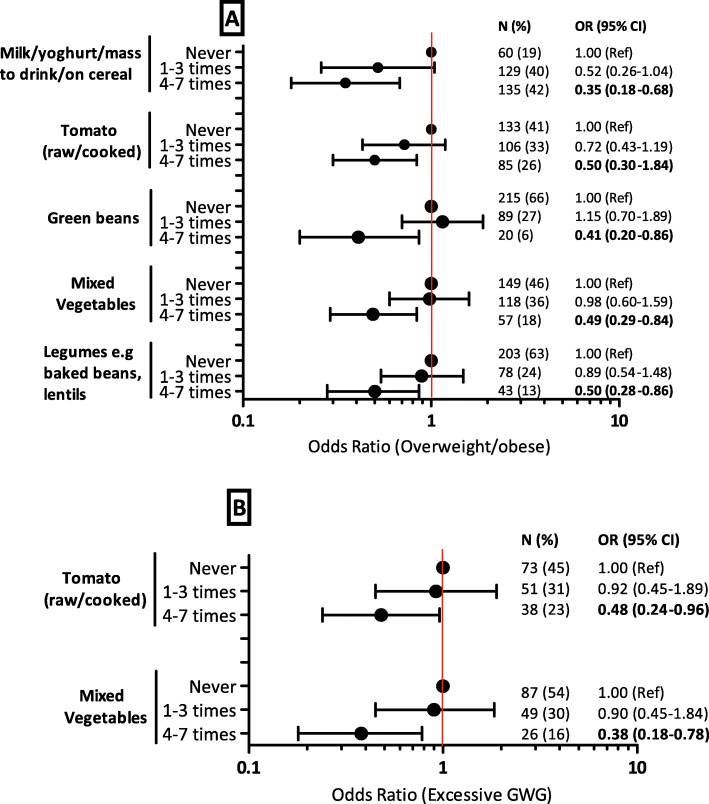
Associations between food consumption frequency and overweight/obesity (**A**) and excessive GWG (**B**) in women with HIV

Figure 4 shows the association between food intake frequency and overweight/obesity (A) and excessive GWG (B) in women with HIV (30 food items shown in Additional files 3 and 4). Consumption of milk/yoghurt/maas to drink/on cereals (aOR 0.35, 95% CI 0.18–0.68), tomato (raw/cooked) (aOR 0.50, 95% CI 0.30–0.84), green beans (aOR 0.41, 95% CI 0.20–0.86), mixed vegetables (aOR 0.49, 95% CI 0.29–0.84) and legumes e.g. baked beans, lentils (aOR 0.50, 95% CI 0.28–0.86) for 4–7 days a week was negatively associated with reduced odds of overweight/obesity compared to not consuming any in the past week. Consumption of tomato (raw/cooked) (aOR 0.48, 95% CI 0.24–0.96) and mixed vegetables (aOR 0.38, 95% CI 0.18–0.78) was also associated with reduced odds of excessive GWG compared to not consuming any in the past week.

## Discussion

In this cohort we found that obesity during pregnancy and excessive GWG was common in both women living with and without HIV. Women without HIV who consumed potato (any preparation) and pumpkin/butternut for 1–3 days a week were more likely to be overweight/obese; and milk in tea/coffee more likely to have excessive GWG. Further, those who consumed eggs (any) for 1–3 days a week were less likely to be overweight/obese; and peanut and nuts for 4–7 days a week less likely to have excessive GWG. Women with HIV who consumed milk/yoghurt/maas to drink/on cereals, tomato (raw/cooked), green beans, mixed vegetables and legumes e.g. baked beans, lentils for 4–7 days a week were less likely to be overweight/obese. Further, those who consumed tomato (raw/cooked) and mixed vegetables for 4–7 days a week were less likely to have excessive GWG. Despite similar trends of obesity and GWG in women living both with and without HIV, positive associations with frequency of food intake were only observed in women without HIV. These data suggest that interventions targeted at modifying food-based dietary intake may play a significant role in reducing overweight/obesity and excessive GWG in pregnant women, particularly those living without HIV.

Obesity is a major public health concern in women as it has detrimental effects on women’s health as well as that of their offspring [7, 10]. The global nutrition transition, particularly increased consumption of an energy-dense diet, has been recognized as one of the major contributing factors to the constant rise in obesity. We found high consumption of potato (any preparation) in our cohort and this was associated with increased risk of overweight/obesity; women with excessive GWG were more likely to consume potato (any preparation) for 4–7 days a week compared to those with adequate GWG. Other authors have reported associations between starch and obesity noting that the nature of the relationship depends on the type of starch being consumed [25–27]. Starchy foods that are rapidly digestible (high-glycaemic index) have worse health effects than those that are slowly digestible, as the former promote postprandial systemic hypoglycaemia accompanied by hunger and overeating [25, 26]. Potatoes have a high-glycaemic index [28, 29] and this may explain the mechanisms mediating the observed associations with overweight/obesity in women without HIV. In contrast, sweet potatoes have been shown to have protective effect against obesity due to their slow digestibility accompanied by sustained satiety [30, 31], however this food item was the least consumed starch in our cohort.

Vegetable consumption had more influence on maternal weight in women with HIV compared to those without. In particular, tomato (raw/cooked) and mixed vegetables decreased the odds of overweight/obesity and excessive GWG in women with HIV; while green beans only reduced overweight/obesity. The importance of vegetables in the daily diet is highlighted in the Global Burden of Disease study which found that consumption of diet low in vegetables is among the leading dietary risk factors for mortality, contributing to more than 2% of global deaths [27]. Surprisingly in our study, women without HIV who consumed pumpkin/butternut for 1–3 days a week were more likely to be overweight/obese compared to those who did not consume any. Although not measured, we speculated that this was due to big portion sizes of pumpkin-containing dishes in this setting. This was shown in a study conducted in the Eastern Cape [32], a province from which most women in our cohort come from. Other scholars have reported controversial findings between vegetable consumption and weight, the study that used a 131-item semiquantitative FFQ found no association [33], whereas another study that used 3-item semiquantitative FFQ found a positive association [34]. Notably, vegetable consumption is most effective in maintenance or reduction of weight when it replaces the intake of high-energy dense foods [35], it remains unclear whether this was the case in our cohort as the FFQ used mainly consisted of healthy foods.

Consumption of eggs (any) in women without HIV, and legumes (baked beans, lentils) in women with HIV decreased the likelihood of overweight/obese. In addition, peanut and nuts decreased the odds of excessive GWG in those without HIV. Protein-rich diets, including legumes have been recommended for maintenance of healthy weight due to their high satiety effect (high fibre and low glycaemic index) which may favour energy intake control [36]. Specifically, several studies are in agreement with our results as they found that traditional diets containing legumes reduce the risk of excessive GWG, including having beneficial weight loss effects for those with obesity [37–39]. Dairy products, including milk have also been reported to have a negative association with weight in women who initially had normal weight [40]. In our study, we obtained similar results as women with HIV who consumed milk/yoghurt/maas to drink/on cereals were less likely to experience excessive weight gain, however those without HIV who consumed milk in tea/coffee were more likely. These conflicting results warrant further study on the relationship between dairy products and maternal weight in settings where HIV is prevalent.

Despite similar consumption frequencies for potato, pumpkin/butternut and milk in tea/coffee in both women with and without HIV, positive associations with overweight/obesity and/or GWG were not observed in women with HIV, regardless of length of time on ART. This suggests that other than food intake, there may be factors influencing obesity and GWG in this group. Several studies reporting obesity in women with HIV attributed this trend to ‘return to health’ phenomenon as a results of access to early treatment due to international policy shifts; as well as exposure to the same obesogenic environment as others [41, 42]. However, factors specific to HIV infection and treatment regimens have also been implicated in influencing the vulnerability of people living with HIV to increased obesity [43]. Despite the growing number of studies reporting obesity in women with HIV in the general population [3, 5, 44, 45], there are limited data on GWG trends in this group. One South African study reported that women with HIV experience greater inadequate GWG [39], however, we found high prevalence of excessive GWG in our cohort. These differences may be partly attributed to a larger proportion of women who initiated ART pre-pregnancy in our cohort, enabling us to reflect recent trends in weight gain in these women.

Our data should be interpreted with caution since a chance of false-positives due to multiple testing conducted cannot be excluded. However, alpha correction approaches increase a chance of false-negatives due to very stringent significance levels. A non-quantitative FFQ was used and therefore quantity/exact consumption of foods reported was not assessed. Furthermore, the 30 food items investigated did not include some of the most common foods such as staple food maize meal. However, we were able to ascertain broader dietary intake trends that should be a key focus for future studies aimed at assessing quantity of food intake and obesity in pregnant women. In conclusion, potato (any preparation) and pumpkin/butternut consumption were associated with increased odds of overweight/obesity, while milk in tea/coffee consumption increased the odds of excessive GWG in women without HIV. Studies investigating potato (any preparation) and pumpkin/butternut portion sizes associated with obesity in pregnant women without HIV are needed. Mechanisms underlying high levels of obesity and GWG in women with HIV unrelated to food intake require further investigation.

## Supplementary Information

**Additional file 1. **Frequencies of BMI (**A**) and GWG (**B**) categories overall and stratified by HIV status.

**Additional file 2.** Frequencies of individual item food-based dietary consumption, overall and stratified by maternal HIV status, BMI and GWG category.

**Additional file 3.** Association between food consumption frequency and maternal BMI, overall and stratified by HIV status.

**Additional file 4.** Association between food consumption frequency and maternal GWG, overall and stratified by HIV status.

## Data Availability

All data generated or analysed during this study are included in this published article (and its supplementary information files). In addition, the datasets used and/or analysed during the current study are available from the corresponding author on reasonable request.
