# Supplementary figures and images for: Association between food intake and obesity in pregnant women living with and without HIV in Cape Town, South Africa: a prospective cohort study
Source: BMC Public Health. 2021 Aug 4;21:1504. doi: 10.1186/s12889-021-11566-2 (PMC8335890; doi:10.1186/s12889-021-11566-2)

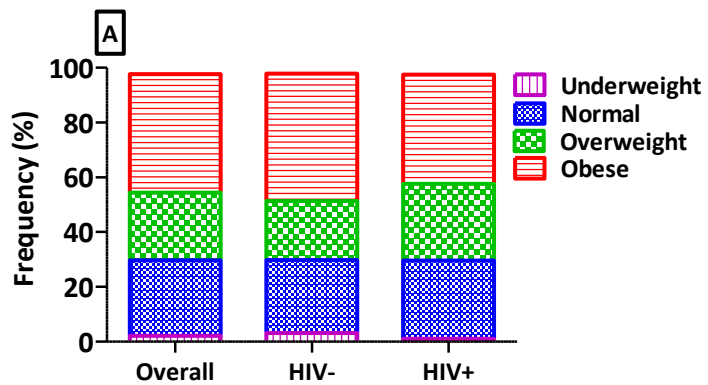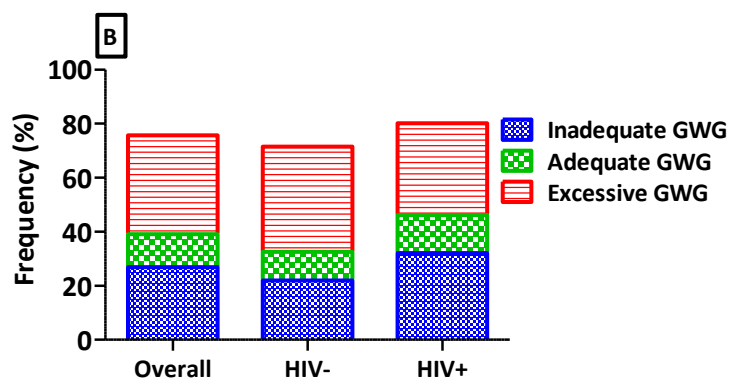

Supplement: Supplementary file 1 — Additional file 1. Frequencies of BMI (A) and GWG (B) categories overall and stratified by HIV status. [file 12889_2021_11566_MOESM1_ESM.pdf]
