# Supplementary material for: Association between food intake and obesity in pregnant women living with and without HIV in Cape Town, South Africa: a prospective cohort study
Source: BMC Public Health. 2021 Aug 4;21:1504. doi: 10.1186/s12889-021-11566-2 (PMC8335890; doi:10.1186/s12889-021-11566-2)
Supplement: Supplementary file 2 — Additional file 2. Frequencies of individual item food-based dietary consumption, overall and stratified by maternal HIV status, BMI and GWG category. [file 12889_2021_11566_MOESM2_ESM.docx]

Additional file 2. Frequencies of individual item food-based dietary consumption, overall and stratified by maternal HIV status, BMI and GWG category

|  |  | HIV status | |  | BMI | | |  | GWG | | |  |
| --- | --- | --- | --- | --- | --- | --- | --- | --- | --- | --- | --- | --- |
| Consumption frequency  (per 7 days) | Overall  N (%) = 989 | Without HIV  N (%) = 510 | With HIV-  N (%) = 479 | p value | Normal  N (%) = 294 | Overweight  N (%) = 244 | Obese  N (%) = 427 | p value | Inadequate  N (%) = 264 | Adequate  N (%) = 121 | Excessive  N (%) = 361 | p-value |
| **Starch** |  |  |  |  |  |  |  |  |  |  |  |  |
| Brown/whole wheat bread/rolls  Never  1-3 days  4-7 days  Missing | 112 (11)  305 (31)  569 (58)  3 (0.3) | 60 (12)  165 (32)  283 (55)  2 (0.4) | 52 (11)  140 (29)  286 (60)  1 (0.2) | 0.422 | 35 (12)  80 (27)  177 (61)  2 (0.7) | 33 (14)  69 (28)  142 (58)  0 | 43 (10)  148 (35)  235 (55)  1 (0.2) | 0.177 | 29 (11)  81 (31)  154 (58)  2 (0.8) | 15 (12)  28 (23)  78 (64)  0 | 40 (11)  105 (29)  216 (60)  1 (0.3) | 0.667 |
| Breakfast cereal (instant)  Never  1-3 days  4-7 days  Missing | 502 (51)  219 (22)  265 (27)  3 (0.3) | 250 (49)  108 (21)  150 (29)  2 (0.4) | 252 (53)  111 (23)  115 (24)  1 (0.2) | 0.152 | 147 (50)  65 (22)  80 (27)  2 (0.7) | 124 (51)  51 (21)  69 (28)  0 | 224 (52)  97 (23)  105 (25)  1 (0.2) | 0.849 | 138 (52)  66 (25)  60 (24)  2 (0.8) | 61 (50)  23 (19)  37 (31)  0 | 189 (52)  71 (20)  101 (28)  1 (0.3) | 0.291 |
| Oats porridge  Never  1-3 days  4-7 days  Missing | 651 (66)  175 (18)  159 (16)  4 (0.4) | 334 (65)  94 (18)  80 (16)  2 (0.4) | 317 (66)  81 (17)  79 (16)  2 (0.4) | 0.802 | 197 (67)  50 (17)  45 (15)  2 (0.7) | 165 (68)  36 (15)  42 (17)  1 (0.4) | 269 (63)  86 (20)  71 (17)  1 (0.2) | 0.450 | 185 (70)  43 (16)  36 (14)  2 (0.8) | 78 (64)  18 (15)  25 (21)  0 | 229 (63)  67 (19)  65 (18)  1 (0.3) | 0.297 |
| Sweet potato  Never  1-3 days  4-7 days  Missing | 789 (80)  118 (12)  79 (8)  3 (0.3) | 410 (80)  64 (13)  34 (7)  2 (0.4) | 379 (79)  54 (11)  45 (9)  1 (0.2) | 0.261 | 222 (76)  43 (15)  27 (9)  2 (0.7) | 189 (77)  27 (11)  28 (11)  0 | 358 (84)  46 (11)  22 (5)  1 (0.2) | **0.013** | 216 (82)  29 (11)  19 (7)  2 (0.8) | 101 (83)  12 (10)  8 (7)  0 | 282 (78)  42 (12)  37 (10)  1 (0.3) | 0.558 |
| Potato (any preparation)  Never  1-3 days  4-7 days  Missing | 115 (12)  348 (35)  523 (53)  3 (0.3) | 59 (12)  183 (36)  266 (52)  2 (0.4) | 56 (12)  165 (34)  257 (54)  1 (0.2) | 0.882 | 42 (14)  94 (32)  156 (53)  2 (0.7) | 27 (11)  93 (38)  124 (51)  0 | 42 (10)  153 (36)  231 (54)  1 (0.2) | 0.301 | 35 (13)  104 (39)  125 (47)  2 (0.8) | 17 (14)  39 (32)  65 (54)  0 | 38 (11)  108 (30)  215 (60)  1 (0.3) | **0.045** |
|  |  |  |  |  |  |  |  |  |  |  |  |  |
| **Protein** |  |  |  |  |  |  |  |  |  |  |  |  |
| Red meat (any)  Never  1-3 days  4-7 days  Missing | 509 (51)  326 (33)  151 (15)  3 (0.3) | 258 (51)  177 (35)  73 (14)  2 (0.4) | 251 (52)  149 (31)  78 (16)  1 (0.2) | 0.416 | 156 (53)  93 (32)  43 (15)  2 (0.7) | 124 (51)  82 (34)  38 (16)  0 | 216 (51)  145 (34)  65 (15)  1 (0.2) | 0.962 | 144 (55)  92 (35)  28 (11)  2 (0.8) | 66 (55)  33 (27)  22 (18)  0 | 173 (48)  124 (34)  64 (18)  1 (0.3) | 0.059 |
| Organ meat e.g. liver  Never  1-3 days  4-7 days  Missing | 638 (65)  224 (23)  124 (13)  3 (0.3) | 334 (65)  116 (23)  58 (11)  2 (0.4) | 304 (63)  108 (23)  66 (14)  1 (0.2) | 0.522 | 190 (65)  61 (21)  41 (14)  2 (0.7) | 154 (63)  60 (25)  30 (12)  0 | 278 (65)  99 (23)  49 (11)  1 (0.2) | 0.760 | 173 (66)  64 (24)  27 (10)  2 (0.8) | 77 (64)  23 (19)  21 (17)  0 | 244 (68)  75 (21)  42 (12)  1 (0.3) | 0.270 |
| Chicken (any)  Never  1-3 days  4-7 days  Missing | 155 (16)  356 (36)  475 (48)  3 (0.3) | 82 (16)  188 (37)  338 (47)  2 (0.4) | 73 (15)  168 (35)  237 (49)  1 (0.2) | 0.692 | 44 (15)  112 (38)  136 (47)  2 (0.7) | 41 (17)  76 (31)  127 (52)  0 | 67 (16)  154 (36)  205 (48)  1 (0.2) | 0.535 | 57 (22)  94 (36)  113 (43)  2 (0.8) | 20 (17)  41 (34)  60 (50)  0 | 51 (14)  116 (32)  194 (54)  1 (0.3) | 0.055 |
| Tinned fish  Never  1-3 days  4-7 days  Missing | 615 (62)  225 (23)  146 (15)  3 (0.3) | 325 (64)  105 (21)  78 (15)  2 (0.4) | 290 (61)  120 (25)  68 (14)  1 (0.2) | 0.251 | 189 (65)  62 (21)  41 (14)  2 (0.7) | 140 (57)  67 (27)  37 (15)  0 | 273 (64)  89 (21)  64 (15)  1 (0.2) | 0.297 | 164 (62)  65 (25)  35 (13)  2 (0.8) | 78 (64)  27 (22)  16 (13)  0 | 238 (66)  74 (21)  49 (14)  1 (0.3) | 0.822 |
| Eggs (any)  Never  1-3 days  4-7 days  Missing | 339 (34)  362 (37)  285 (29)  3 (0.3) | 156 (31)  185 (36)  167 (33)  2 (0.4) | 183 (38)  177 (37)  118 (25)  1 (0.2) | **0.007** | 95 (33)  114 (39)  83 (28)  2 (0.7) | 87 (36)  84 (34)  73 (30)  0 | 153 (36)  155 (37)  118 (28)  1 (0.2) | 0.792 | 108 (41)  92 (35)  64 (24)  2 (0.8) | 40 (33)  41 (34)  40 (33)  0 | 120 (33)  136 (38)  105 (29)  1 (0.3) | 0.204 |
|  |  |  |  |  |  |  |  |  |  |  |  |  |
| **Dairy** |  |  |  |  |  |  |  |  |  |  |  |  |
| Milk/yoghurt/maas to drink/on cereals  Never  1-3 days  4-7 days  Missing | 161 (16)  372 (38)  453 (46)  3 (0.3) | 86 (17)  187 (37)  235 (46)  2 (0.4) | 75 (16)  185 (39)  218 (46)  1 (0.2) | 0.784 | 42 (14)  110 (38)  140 (48)  2 (0.7) | 42 (17)  88 (36)  114 (47)  0 | 76 (18)  163 (38)  187 (44)  1 (0.2) | 0.701 | 48 (18)  106 (40)  110 (42)  2 (0.8) | 20 (17)  37 (31)  64 (53)  0 | 58 (16)  129 (36)  174 (48)  1 (0.3) | 0.267 |
| Milk in tea/coffee  Never  1-3 days  4-7 days  Missing | 442 (45)  221 (22)  323 (33)  3 (0.3) | 238 (47)  106 (21)  164 (32)  2 (0.4) | 204 (43)  115 (24)  159 (33)  1 (0.2) | 0.342 | 138 (47)  58 (20)  96 (33)  2 (0.7) | 99 (41)  54 (22)  91 (37)  0 | 196 (46)  101 (24)  129 (30)  1 (0.2) | 0.290 | 122 (46)  68 (26)  74 (28)  2 (0.8) | 59 (49)  16 (13)  46 (38)  0 | 161 (45)  73 (20)  127 (35)  1 (0.3) | **0.038** |
| Cheese (except cottage)  Never  1-3 days  4-7 days  Missing | 575 (58)  234 (24)  177 (18)  3 (0.3 | 308 (60)  112 (22)  88 (17)  2 (0.4) | 267 (56)  122 (25)  89 (19)  1 (0.2) | 0.294 | 180 (62)  66 (23)  46 (16)  2 (0.7) | 138 (57)  60 (25)  46 (19)  0 | 244 (57)  103 (24)  79 (19)  1 (0.2) | 0.736 | 157 (59)  56 (21)  51 (19)  2 (0.8) | 66 (55)  35 (29)  20 (17)  0 | 219 (61)  83 (23)  59 (16)  1 (0.3) | 0.468 |
|  |  |  |  |  |  |  |  |  |  |  |  |  |
| **Fruits** |  |  |  |  |  |  |  |  |  |  |  |  |
| Citrus fruit e.g. orange  Never  1-3 days  4-7 days  Missing | 464 (47)  326 (33)  195 (20)  4 (0.4) | 254 (50)  163 (32)  91 (18)  2 (0.4) | 210 (45)  163 (34)  104 (21)  2 (0.4) | 0.131 | 151 (52)  85 (29)  56 (19)  2 (0.7) | 120 (49)  78 (32)  45 (18)  1 (0.4) | 188 (44)  153 (36)  85 (20)  1 (0.2) | 0.294 | 110 (42)  113 (43)  41 (16)  2 (0.8) | 55 (45)  33 (27)  33 (27)  0 | 190 (53)  102 (28)  68 (19)  2 (0.6) | **<0.001** |
| Pure orange/guava juice  Never  1-3 days  4-7 days  Missing | 571 (58)  267 (27)  148 (15)  3 (0.3) | 289 (57)  145 (28)  74 (15)  2 (0.4) | 282 (59)  122 (25)  74 (15)  1 (0.2) | 0.561 | 176 (60)  74 (25)  42 (14)  2 (0.7) | 130 (53)  75 (31)  39 (16)  0 | 254 (29)  109 (26)  63 (15)  1 (0.2) | 0.478 | 154 (58)  72 (27)  38 (14)  2 (0.8) | 65 (54)  31 (26)  25 (21)  0 | 217 (60)  92 (25)  52 (14)  1 (0.3) | 0.491 |
| Banana  Never  1-3 days  4-7 days  Missing | 253 (26)  414 (42)  318 (32)  4 (0.4) | 135 (26)  209 (41)  164 (32)  2 (0.4) | 118 (25)  205 (43)  154 (32)  2 (0.4) | 0.771 | 74 (25)  127 (43)  91 (31)  2 (0.7) | 56 (23)  96 (39)  92 (38)  0 | 118 (28)  183 (43)  124 (29)  2 (0.5) | 0.223 | 64 (24)  124 (47)  76 (29)  2 (0.8) | 36 (30)  44 (36)  41 (34)  0 | 89 (25)  146 (44)  126 (35)  1 (0.3) | 0.230 |
| Mangoes  Never  1-3 days  4-7 days  Missing | 836 (85)  104 (11)  45 (4)  4 (0.4) | 434 (85)  50 (10)  24 (5)  2 (0.4) | 402 (84)  54 (11)  21 (4)  2 (0.4) | 0.739 | 244 (84)  35 (12)  13 (4)  2 (0.7) | 200 (82)  31 (13)  13 (5)  0 | 373 (87)  35 (8)  17 (4)  2 (0.5) | 0.273 | 217 (82)  37 (14)  10 (4)  2 (0.8) | 105 (87)  11 (9)  5 (4)  0 | 326 (90)  24 (7)  11 (3)  1 (0.3) | **0.037** |
| Apples/pears  Never  1-3 days  4-7 days  Missing | 215 (22)  426 (43)  344 (35)  4 (0.4) | 103 (20)  217 (43)  188 (37)  2 (0.4) | 112 (23)  209 (44)  156 (33)  2 (0.4) | 0.282 | 55 (19)  132 (45)  105 (36)  2 (0.7) | 53 (22)  102 (42)  89 (36)  0 | 106 (25)  178 (42)  141 (33)  2 (0.5) | 0.392 | 70 (27)  110 (42)  84 (32)  2 (0.8) | 27 (22)  48 (40)  46 (38)  0 | 79 (22)  148 (41)  134 (37)  1 (0.3) | 0.548 |
| Avocado  Never  1-3 days  4-7 days  Missing | 671 (68)  184 (19)  130 (13)  4 (0.4) | 356 (70)  89 (17)  63 (12)  2 (0.4) | 315 (66)  95 (20)  67 (14)  2 (0.4) | 0.397 | 204 (70)  50 (17)  38 (13)  2 (0.7) | 155 (64)  52 (21)  37 (15)  0 | 296 (70)  77 (18)  52 (12)  2 (0.5) | 0.492 | 183 (69)  55 (21)  26 (10)  2 (0.8) | 76 (63)  24 (20)  21 (17)  0 | 159 (72)  55 (15)  47 (13)  1 (0.3) | 0.099 |
|  |  |  |  |  |  |  |  |  |  |  |  |  |
| **Vegetables** |  |  |  |  |  |  |  |  |  |  |  |  |
| Broccoli  Never  1-3 days  4-7 days  Missing | 862 (87)  78 (8)  46 (5)  3 (0.3) | 451 (88)  35 (7)  22 (4)  2 (0.4) | 411 (86)  43 (9)  24 (5)  1 (0.2) | 0.396 | 258 (88)  24 (8)  10 (3)  2 (0.7) | 199 (82)  29 (12)  16 (7)  0 | 383 (90)  24 (6)  19 (4)  1 (0.2) | **0.019** | 231 (88)  21 (8)  12 (5)  2 (0.8) | 104 (86)  12 (10)  5 (4)  0 | 319 (88)  27 (7)  15 (4)  1 (0.3) | 0.939 |
| Spinach (including morogo)  Never  1-3 days  4-7 days  Missing | 502 (51)  325 (33)  159 (16)  3 (0.3) | 277 (54)  151 (30)  80 (16)  2 (0.4) | 225 (47)  174 (36)  79 (16)  1 (0.2) | **0.047** | 150 (51)  95 (33)  47 (16)  2 (0.7) | 119 (49)  86 (35)  39 (16)  0 | 219 (51)  137 (32)  70 (16)  1 (0.2) | 0.945 | 116 (44)  102 (39)  46 (17)  2 (0.8) | 58 (48)  42 (35)  21 (17)  0 | 202 (56)  98 (27)  61 (17)  1 (0.3) | **0.026** |
| Carrots  Never  1-3 days  4-7 days  Missing | 229 (23)  409 (41)  348 (35)  3 (0.3) | 126 (25)  200 (39)  182 (36)  2 (0.4) | 103 (22)  209 (44)  166 (35)  1 (0.2) | 0.311 | 70 (24)  118 (40)  104 (36)  2 (0.7) | 57 (23)  96 (39)  91 (37)  0 | 96 (22)  185 (43)  145 (34)  1 (0.2) | 0.849 | 66 (25)  106 (40)  92 (35)  2 (0.8) | 31 (26)  50 (41)  40 (33)  0 | 84 (23)  143 (40)  134 (37)  1 (0.3) | 0.930 |
| Tomato (raw/cooked)  Never  1-3 days  4-7 days  Missing | 382 (39)  319 (32)  285 (29)  3 (0.3) | 200 (39)  159 (31)  149 (29)  2 (0.4) | 182 (38)  160 (33)  136 (28)  1 (0.2) | 0.766 | 106 (36)  99 (34)  87 (30)  2 (0.7) | 89 (36)  85 (35)  70 (29)  0 | 180 (42)  125 (29)  121 (28)  1 (0.2) | 0.393 | 99 (38)  94 (36)  71 (27)  2 (0.8) | 48 (40)  31 (26)  42 (35)  0 | 148 (41)  111 (31)  102 (28)  1 (0.3) | 0.277 |
| Green beans  Never  1-3 days  4-7 days  Missing | 697 (70)  222 (22)  67 (7)  3 (0.3) | 378 (74)  102 (20)  28 (5)  2 (0.4) | 319 (67)  120 (25)  39 (8)  1 (0.2) | **0.025** | 204 (70)  60 (21)  28 (10)  2 (0.7) | 175 (72)  52 (21)  17 (7)  0 | 296 (70)  108 (25)  22 (5)  1 (0.2) | 0.132 | 177 (67)  69 (26)  18 (7)  2 (0.8) | 85 (70)  25 (21)  11 (9)  0 | 272 (75)  69 (19)  20 (6)  1 (0.3) | 0.146 |
| Green peas  Never  1-3 days  4-7 days  Missing | 676 (68)  231 (23)  79 (8)  3 (0.3) | 356 (70)  114 (22)  38 (7)  2 (0.4) | 320 (67)  117 (24)  41 (9)  1 (0.2) | 0.560 | 208 (71)  56 (19)  28 (10)  2 (0.7) | 167 (68)  59 (24)  18 (7)  0 | 281 (66)  114 (27)  31 (7)  1 (0.2) | 0.183 | 179 (68)  67 (25)  18 (7)  2 (0.8) | 77 (64)  31 (26)  13 (11)  0 | 262 (72)  72 (20)  27 (7)  1 (0.3) | 0.248 |
| Mixed vegetables  Never  1-3 days  4-7 days  Missing | 445 (45)  333 (34)  208 (21)  3 (0.3) | 231 (45)  167 (33)  110 (22)  2 (0.4) | 214 (45)  166 (35)  98 (20)  1 (0.2) | 0.806 | 135 (46)  87 (30)  70 (24)  2 (0.7) | 114 (47)  82 (34)  48 (20)  0 | 184 (43)  155 (36)  87 (20)  1 (0.2) | 0.375 | 114 (43)  96 (36)  54 (20)  2 (0.8) | 55 (45)  31 (26)  35 (29)  0 | 177 (49)  117 (32)  67 (19)  1 (0.3) | 0.066 |
| Pumpkin/butternut  Never  1-3 days  4-7 days  Missing | 392 (40)  305 (31)  288 (29)  4 (0.4) | 208 (41)  150 (29)  149 (29)  3 (0.6) | 184 (38)  155 (32)  139 (29)  1 (0.2) | 0.593 | 136 (47)  75 (26)  81 (28)  2 (0.7) | 93 (38)  87 (26)  64 (26)  0 | 156 (37)  134 (31)  135 (32)  2 (0.4) | **0.028** | 105 (40)  88 (33)  71 (27)  2 (0.8) | 49 (41)  37 (31)  35 (29)  0 | 151 (42)  106 (29)  103 (29)  2 (0.6) | 0.889 |
|  |  |  |  |  |  |  |  |  |  |  |  |  |
| **Legumes** |  |  |  |  |  |  |  |  |  |  |  |  |
| Legumes e.g. baked beans, lentils  Never  1-3 days  4-7 days  Missing | 611 (62)  239 (24)  136 (14)  3 (0.3) | 319 (63)  127 (25)  62 (12)  2 (0.4) | 292 (61)  112 (23)  74 (15)  1 (0.2) | 0.319 | 179 (61)  70 (24)  43 (15)  2 (0.7) | 155 (64)  54 (22)  35 (14)  0 | 264 (62)  108 (25)  54 (13)  1 (0.2) | 0.843 | 162 (61)  68 (26)  34 (13)  2 (0.8) | 77 (64)  29 (24)  15 (12)  0 | 232 (64)  80 (22)  49 (14)  1 (0.3) | 0.885 |
| Peanut and nuts  Never  1-3 days  4-7 days  Missing | 748 (76)  138 (14)  100 (10)  3 (0.3) | 387 (76)  70 (14)  51 (10)  2 (0.4) | 361 (75)  68 (14)  49 (10)  1 (0.2) | 0.970 | 220 (75)  42 (14)  30 (10)  2 (0.7) | 186 (76)  32 (13)  26 (11)  0 | 325 (76)  63 (15)  38 (9)  1 (0.2) | 0.922 | 199 (75)  36 (14)  29 (11)  2 (0.8) | 97 (80)  10 (8)  14 (12)  0 | 286 (79)  45 (12)  30 (8)  1 (0.3) | 0.411 |
|  |  |  |  |  |  |  |  |  |  |  |  |  |
| **Fats/oils** |  |  |  |  |  |  |  |  |  |  |  |  |
| Soft margarine (tub)  Never  1-3 days  4-7 days  Missing | 333 (34)  267 (27)  386 (39)  3 (0.3) | 173 (34)  137 (27)  198 (39)  2 (0.4) | 160 (33)  130 (27)  188 (39)  1 (0.2) | 0.981 | 99 (34)  79 (27)  114 (39)  2 (0.7) | 84 (34)  70 (29)  90 (37)  0 | 141 (33)  111 (26)  174 (41)  1 (0.2) | 0.892 | 95 (36)  77 (29)  92 (36)  2 (0.8) | 48 (40)  23 (19)  50 (41)  0 | 112 (31)  87 (24)  162 (45)  1 (0.3) | **0.044** |
