# Supplementary material for: Association between food intake and obesity in pregnant women living with and without HIV in Cape Town, South Africa: a prospective cohort study
Source: BMC Public Health. 2021 Aug 4;21:1504. doi: 10.1186/s12889-021-11566-2 (PMC8335890; doi:10.1186/s12889-021-11566-2)
Supplement: Supplementary file 3 — Additional file 3. Association between food consumption frequency and maternal BMI, overall and stratified by HIV status. [file 12889_2021_11566_MOESM3_ESM.docx]

| Additional file 3. Association between food consumption frequency and maternal BMI, overall and stratified by HIV status |
| --- |

|  |  |  | | | **HIV status** | | | | | |
| --- | --- | --- | --- | --- | --- | --- | --- | --- | --- | --- |
|  |  | **Overall** | | | **Without HIV** | | | **With HIV** | | |
|  |  |  | **Unadjusted** | **Adjusted** |  | **Unadjusted** | **Adjusted** |  | **Unadjusted** | **Adjusted** |
| **Food group** | **BMI (kg/m^2^)** | **Total**  **N (%) = 963** | **OR (95% CI)** | **aOR (95% CI)** | **Total**  **N (%) = 497** | **OR (95% CI)** | **aOR (95% CI)** | **Total**  **N (%) = 466** | **OR (95% CI)** | **aOR (95% CI)** |
| **STARCH**  Brown/whole wheat bread/rolls |  |  |  |  |  |  |  |  |  |  |
|  | *Overweight/Obese* | 671 (70) |  |  | 347 (70) |  |  | 324 (70) |  |  |
|  | Never | 76 (11) | 1.00 (Ref) | 1.00 (Ref) | 40 (12) | 1.00 (Ref) | 1.00 (Ref) | 36 (11) | 1.00 (Ref) | 1.00 (Ref) |
|  | 1-3 days | 217 (32) | 1.25 (0.78-2.01) | 1.17 (0.72-1.91) | 112 (32) | 1.12 (0.60-2.11) | 1.06 (0.55-2.04) | 105 (32) | 1.46 (0.70-3.02) | 1.41 (0.66-2.99) |
|  | 4-7 days | 378 (56) | 0.98 (0.63-1.52) | 0.91 (0.58-1.44) | 195 (56) | 1.22 (0.67-2.21) | 1.18 (0.64-2.17) | 183 (56) | 0.79 (0.41-1.51) | 0.71 (0.36-1.40) |
| Breakfast cereal (instant) |  |  |  |  |  |  |  |  |  |  |
|  | *Overweight/Obese* | 671 (70) |  |  | 347 (70) |  |  | 324 (70) |  |  |
|  | Never | 349 (52) | 1.00 (Ref) | 1.00 (Ref) | 174 (50) | 1.00 (Ref) | 1.00 (Ref) | 175 (54) | 1.00 (Ref) | 1.00 (Ref) |
|  | 1-3 days | 148 (22) | 0.96 (0.68-1.36) | 0.95 (0.66-1.36) | 74 (21) | 0.97 (0.59-1.60) | 0.95 (0.56-1.60) | 74 (23) | 0.95 (0.58-1.55) | 0.95 (0.57-1.58) |
|  | 4-7 days | 174 (26) | 0.92 (0.66-1.27) | 0.94 (0.66-1.32) | 99 (29) | 0.92 (0.59-1.44) | 0.94 (0.59-1.50) | 75 (23) | 0.91 (0.56-1.47) | 0.88 (0.53-1.48) |
| Oats porridge |  |  |  |  |  |  |  |  |  |  |
|  | *Overweight/Obese* | 671 (70) |  |  | 347 (70) |  |  | 324 (70) |  |  |
|  | Never | 435 (65) | 1.00 (Ref) | 1.00 (Ref) | 225 (65) | 1.00 (Ref) | 1.00 (Ref) | 210 (65) | 1.00 (Ref) | 1.00 (Ref) |
|  | 1-3 days | 122 (18) | 1.11 (0.76-1.60) | 1.08 (0.74-1.58) | 62 (18) | 0.88 (0.54-1.44) | 0.86 (0.52-1.42) | 60 (19) | 1.47 (0.83-2.60) | 1.43 (0.79-2.60) |
|  | 4-7 days | 113 (17) | 1.14 (0.77-1.67) | 1.13 (0.76-1.70) | 60 (17) | 1.32 (0.75-2.31) | 1.37 (0.77-2.46) | 53 (16) | 0.99 (0.58-1.69) | 0.95 (0.54-1.66) |
| Sweet potato |  |  |  |  |  |  |  |  |  |  |
|  | *Overweight/Obese* | 671 (70) |  |  | 347 (70) |  |  | 324 (70) |  |  |
|  | Never | 548 (83) | 1.00 (Ref) | 1.00 (Ref) | 288 (83) | 1.00 (Ref) | 1.00 (Ref) | 260 (80) | 1.00 (Ref) | 1.00 (Ref) |
|  | 1-3 days | 73 (11) | 0.69 (0.46-1.03) | 0.70 (0.46-1.07) | 37 (11) | **0.56 (0.33-0.97)** | 0.60 (0.33-1.09) | 36 (11) | 0.88 (0.47-1.63) | 0.85 (0.46-1.58) |
|  | 4-7 days | 50 (7) | 0.75 (0.46-1.23) | 0.74 (0.43-1.25) | 22 (6) | 0.87 (0.40-1.90) | 0.95 (0.42-2.13) | 28 (9) | 0.68 (0.36-1.30) | 0.65 (0.32-1.32) |
| Potato (any preparation) |  |  |  |  |  |  |  |  |  |  |
|  | *Overweight/Obese* | 671 (70) |  |  | 347 (70) |  |  | 324 (70) |  |  |
|  | Never | 69 (10) | 1.00 (Ref) | 1.00 (Ref) | 33 (10) | 1.00 (Ref) | 1.00 (Ref) | 36 (11) | 1.00 (Ref) | 1.00 (Ref) |
|  | 1-3 days | 247 (37) | **1.60 (1.02-2.51)** | **1.72 (1.08-2.74)** | 131 (38) | **1.86 (1.01-3.48)** | **1.98 (1.02-3.84)** | 116 (36) | 1.36 (0.71-2.62) | 1.48 (0.76-2.88) |
|  | 4-7 days | 355 (53) | 1.39 (0.90-2.12) | 1.44 (0.93-2.25) | 183 (53) | 1.64 (0.90-2.97) | 1.63 (0.87-3.05) | 172 (53) | 1.16 (0.63-2.16) | 1.24 (0.66-2.35) |
| **PROTEIN** |  |  |  |  |  |  |  |  |  |  |
| Red meat (any) | *Overweight/Obese* | 671 (70) |  |  | 347 (70) |  |  | 324 (70) |  |  |
|  | Never | 341 (51) | 1.00 (Ref) | 1.00 (Ref) | 175 (50) | 1.00 (Ref) | 1.00 (Ref) | 166 (51) | 1.00 (Ref) | 1.00 (Ref) |
|  | 1-3 days | 227 (34) | 1.12 (0.82-1.52) | 1.19 (0.87-1.64) | 118 (34) | 0.94 (0.62-1.42) | 1.04 (0.68-1.61) | 109 (34) | 1.38 (0.87-2.19) | 1.43 (0.89-2.32) |
|  | 4-7 days | 103 (15) | 1.10 (0.73-1.64) | 1.11 (0.72-1.70) | 54 (16) | 1.50 (0.81-2.79) | 1.50 (0.78-2.87) | 49 (15) | 0.85 (0.50-1.49) | 0.86 (0.48-1.53) |
|  |  |  |  |  |  |  |  |  |  |  |
| Organ meat e.g. liver | *Overweight/Obese* | 671 (70) |  |  | 347 (70) |  |  | 324 (70) |  |  |
|  | Never | 433 (65) | 1.00 (Ref) | 1.00 (Ref) | 227 (65) | 1.00 (Ref) | 1.00 (Ref) | 206 (64) | 1.00 (Ref) | 1.00 (Ref) |
|  | 1-3 days | 159 (24) | 1.14 (0.81-1.61) | 1.03 (0.73-1.45) | 83 (24) | 1.22 (0.75-1.97) | 1.14 (0.69-1.86) | 76 (23) | 1.07 (0.66-1.74) | 0.95 (0.58-1.54) |
|  | 4-7 days | 79 (12) | 0.85 (0.56-1.28) | 0.76 (0.49-1.19) | 37 (11) | 0.81 (0.45-1.47) | 0.76 (0.41-1.43) | 42 (13) | 0.87 (0.49-1.56) | 0.77 (0.40-1.46) |
|  |  |  |  |  |  |  |  |  |  |  |
| Chicken (any) | *Overweight/Obese* | 671 (70) |  |  | 347 (70) |  |  | 324 (70) |  |  |
|  | Never | 152 (16) | 1.00 (Ref) | 1.00 (Ref) | 59 (17) | 1.00 (Ref) | 1.00 (Ref) | 49 (15) | 1.00 (Ref) | 1.00 (Ref) |
|  | 1-3 days | 343 (36) | 0.84 (0.55-1.28) | 0.87 (0.56-1.35) | 122 (35) | 0.81 (0.45-1.43) | 0.80 (0.43-1.49) | 109 (34) | 0.88 (0.50-1.62) | 0.97 (0.52-1.81) |
|  | 4-7 days | 332 (50) | 0.99 (0.66-1.49) | 0.95 (0.62-1.44) | 166 (48) | 0.95 (0.54-1.66) | 0.87 (0.48-1.58) | 166 (51) | 1.05 (0.58-1.88) | 1.06 (0.58-1.90) |
|  |  |  |  |  |  |  |  |  |  |  |
| Tinned fish | *Overweight/Obese* | 671 (70) |  |  | 347 (70) |  |  | 324 (70) |  |  |
|  | Never | 413 (62) | 1.00 (Ref) | 1.00 (Ref) | 216 (62) | 1.00 (Ref) | 1.00 (Ref) | 197 (61) | 1.00 (Ref) | 1.00 (Ref) |
|  | 1-3 days | 157 (23) | 1.16 (0.82-1.63) | 1.12 (0.79-1.59) | 79 (23) | 1.65 (0.98-2.78) | 1.57 (0.92-2.67) | 78 (24) | 0.86 (0.54-1.37) | 0.87 (0.54-1.39) |
|  | 4-7 days | 101 (15) | 1.13 (0.75-1.68) | 1.20 (0.79-1.81) | 52 (15) | 1.09 (0.63-1.88) | 1.19 (0.68-2.09) | 49 (15) | 1.17 (0.65-2.14) | 1.23 (0.66-2.29) |
|  |  |  |  |  |  |  |  |  |  |  |
| Eggs (any) | *Overweight/Obese* | 671 (70) |  |  | 347 (70) |  |  | 324 (70) |  |  |
|  | Never | 240 (36) | 1.00 (Ref) | 1.00 (Ref) | 115 (33) | 1.00 (Ref) | 1.00 (Ref) | 125 (39) | 1.00 (Ref) | 1.00 (Ref) |
|  | 1-3 days | 240 (36) | 0.83 (0.60-1.15) | 0.79 (0.56-1.10) | 115 (33) | **0.57 (0.35-0.91)** | **0.52 (0.32-0.86)** | 125 (39) | 1.21 (0.77-1.92) | 1.12 (0.70-1.80) |
|  | 4-7 days | 191 (28) | 0.91 (0.64-1.29) | 0.91 (0.63-1.32) | 117 (34) | 0.86 (0.52-1.42) | 0.88 (0.52-1.50) | 74 (23) | 0.89 (0.54-1.47) | 0.82 (0.48-1.38) |
| **DAIRY** |  |  |  |  |  |  |  |  |  |  |
| Milk/yoghurt/maas to drink/on cereals | *Overweight/Obese* | 671 (70) |  |  | 347 (70) |  |  | 324 (70) |  |  |
|  | Never | 118 (18) | 1.00 (Ref) | 1.00 (Ref) | 58 (17) | 1.00 (Ref) | 1.00 (Ref) | 60 (19) | 1.00 (Ref) | 1.00 (Ref) |
|  | 1-3 days | 252 (38) | 0.82 (0.54-1.24) | 0.69 (0.44-1.06) | 123 (35) | 0.95 (0.55-1.66) | 0.82 (0.46-1.47) | 129 (40) | 0.65 (0.34-1.24) | 0.52 (0.26-1.04) |
|  | 4-7 days | 301 (45) | 0.77 (0.51-1.15) | **0.64 (0.42-0.98)** | 166 (48) | 1.23 (0.71-2.11) | 1.04 (0.59-1.82) | 135 (42) | **0.44 (0.23-0.82)** | **0.35 (0.18-0.68)** |
|  |  |  |  |  |  |  |  |  |  |  |
| Milk in tea/coffee | *Overweight/Obese* | 671 (70) |  |  | 347 (70) |  |  | 324 (70) |  |  |
|  | Never | 295 (44) | 1.00 (Ref) | 1.00 (Ref) | 157 (45) | 1.00 (Ref) | 1.00 (Ref) | 138 (43) | 1.00 (Ref) | 1.00 (Ref) |
|  | 1-3 days | 156 (23) | 1.26 (0.88-1.81) | 1.20 (0.83-1.74) | 76 (22) | 1.31 (0.79-2.19) | 1.36 (0.80-2.30) | 80 (25) | 1.20 (0.72-2.01) | 1.09 (0.64-1.86) |
|  | 4-7 days | 220 (33) | 1.07 (0.78-1.47) | 0.96 (0.69-1.33) | 114 (33) | 1.20 (0.77-1.86) | 1.11 (0.70-1.75) | 106 (33) | 0.95 (0.61-1.50) | 0.84 (0.52-1.34) |
|  |  |  |  |  |  |  |  |  |  |  |
| Cheese (except cottage) | *Overweight/Obese* | 671 (70) |  |  | 347 (70) |  |  | 324 (70) |  |  |
|  | Never | 383 (57) | 1.00 (Ref) | 1.00 (Ref) | 208 (60) | 1.00 (Ref) | 1.00 (Ref) | 175 (54) | 1.00 (Ref) | 1.00 (Ref) |
|  | 1-3 days | 163 (24) | 1.16 (0.83-1.63) | 1.14 (0.80-1.63) | 79 (23) | 1.12 (0.69-1.80) | 1.15 (0.70-1.90) | 84 (26) | 1.21 (0.75-1.95) | 1.16 (0.69-1.93) |
|  | 4-7 days | 125 (19) | 1.28 (0.87-1.87) | 1.28 (0.86-1.91) | 60 (17) | 1.13 (0.66-1.93) | 1.30 (0.75-2.26) | 65 (20) | 1.45 (0.84-2.51) | 1.30 (0.73-2.34) |
| **FRUITS** |  |  |  |  |  |  |  |  |  |  |
| Citrus fruit e.g. orange | *Overweight/Obese* | 671 (70) |  |  | 347 (70) |  |  | 324 (70) |  |  |
|  | Never | 308 (46) | 1.00 (Ref) | 1.00 (Ref) | 171 (49) | 1.00 (Ref) | 1.00 (Ref) | 137 (42) | 1.00 (Ref) | 1.00 (Ref) |
|  | 1-3 days | 232 (35) | 1.34 (0.98-1.84) | 1.29 (0.94-1.79) | 115 (33) | 1.18 (0.76-1.83) | 1.17 (0.75-1.83) | 117 (36) | 1.54 (0.97-2.43) | 1.47 (0.92-2.36) |
|  | 4-7 days | 130 (19) | 1.14 (0.79-1.65) | 1.09 (0.74-1.60) | 61 (18) | 1.08 (0.64-1.84) | 1.08 (0.62-1.89) | 69 (21) | 1.21 (0.72-2.02) | 1.13 (0.66-1.94) |
|  |  |  |  |  |  |  |  |  |  |  |
| Pure orange/guava juice | *Overweight/Obese* | 671 (70) |  |  | 347 (70) |  |  | 324 (70) |  |  |
|  | Never | 384 (57) | 1.00 (Ref) | 1.00 (Ref) | 194 (56) | 1.00 (Ref) | 1.00 (Ref) | 190 (59) | 1.00 (Ref) | 1.00 (Ref) |
|  | 1-3 days | 184 (27) | 1.14 (0.82-1.58) | 1.08 (0.78-1.52) | 100 (29) | 1.13 (0.73-1.76) | 1.09 (0.69-1.72) | 84 (26) | 1.15 (0.72-1.86) | 1.04 (0.63-1.73) |
|  | 4-7 days | 103 (15) | 1.12 (0.75-1.68) | 1.13 (0.74-1.72) | 53 (15) | 1.29 (0.73-2.31) | 1.41 (0.77-2.57) | 50 (15) | 0.98 (0.56-1.72) | 0.93 (0.52-1.67) |
|  |  |  |  |  |  |  |  |  |  |  |
| Banana | *Overweight/Obese* | 671 (70) |  |  | 347 (70) |  |  | 324 (70) |  |  |
|  | Never | 174 (26) | 1.00 (Ref) | 1.00 (Ref) | 91 (26) | 1.00 (Ref) | 1.00 (Ref) | 83 (26) | 1.00 (Ref) | 1.00 (Ref) |
|  | 1-3 days | 280 (42) | 0.94 (0.66-1.32) | 0.89 (0.69-1.40) | 140 (40) | 0.98 (0.62-1.56) | 1.04 (0.64-1.68) | 140 (43) | 0.88 (0.53-1.47) | 0.94 (0.55-1.59) |
|  | 4-7 days | 216 (32) | 1.01 (0.70-1.46) | 1.03 (0.70-1.51) | 116 (33) | 1.27 (0.77-2.12) | 1.42 (0.84-2.42) | 100 (31) | 0.79 (0.46-1.34) | 0.75 (0.43-1.32) |
|  |  |  |  |  |  |  |  |  |  |  |
| Mangoes | *Overweight/Obese* | 671 (70) |  |  | 347 (70) |  |  | 324 (70) |  |  |
|  | Never | 573 (85) | 1.00 (Ref) | 1.00 (Ref) | 304 (88) | 1.00 (Ref) | 1.00 (Ref) | 269 83) | 1.00 (Ref) | 1.00 (Ref) |
|  | 1-3 days | 67 (10) | 0.82 (0.53-1.26) | 0.74 (0.47-1.16) | 29 (8) | 0.62 (0.33-1.14) | 0.60 (0.31-1.17) | 38 (12) | 1.07 (0.57-1.99) | 0.96 (0.51-1.80) |
|  | 4-7 days | 30 (4) | 0.98 (0.50-1.92) | 1.02 (0.53-2.00) | 14 (4) | 0.71 (0.29-1.73) | 0.79 (0.33-1.91) | 16 (5) | 1.44 (0.51-4.02) | 1.41 (0.51-3.92) |
|  |  |  |  |  |  |  |  |  |  |  |
| Apples/pears | *Overweight/Obese* | 671 (70) |  |  | 347 (70) |  |  | 324 (70) |  |  |
|  | Never | 159 (24) | 1.00 (Ref) | 1.00 (Ref) | 79 (23) | 1.00 (Ref) | 1.00 (Ref) | 80 (25) | 1.00 (Ref) | 1.00 (Ref) |
|  | 1-3 days | 281 (42) | 0.74 (0.51-1.07) | 0.70 (0.48-1.03) | 143 (41) | 1.62 (0.36-1.06) | 0.63 (0.36-1.10) | 138 (43) | 0.86 (0.52-1.44) | 0.76 (0.45-1.30) |
|  | 4-7 days | 230 (34) | 0.76 (0.52-1.11) | 0.74 (0.49-1.10) | 125 (36) | 0.68 (0.39-1.18) | 0.71 (0.40-1.26) | 105 (32) | 0.83 (0.49-1.42) | 0.74 (0.42-1.30) |
|  |  |  |  |  |  |  |  |  |  |  |
| Avocado | *Overweight/Obese* | 671 (70) |  |  | 347 (70) |  |  | 324 (70) |  |  |
|  | Never | 452 (67) | 1.00 (Ref) | 1.00 (Ref) | 239 (69) | 1.00 (Ref) | 1.00 (Ref) | 213 (66) | 1.00 (Ref) | 1.00 (Ref) |
|  | 1-3 days | 129 (19) | 1.16 (0.81-1.68) | 1.04 (0.71-1.53) | 64 (18) | 1.28 (0.76-2.17) | 1.11 (0.63-1.96) | 65 (20) | 1.06 (0.64-1.77) | 0.99 (0.58-1.69) |
|  | 4-7 days | 89 (13) | 1.06 (0.70-1.60) | 0.99 (0.64-1.54) | 44 (13) | 1.91 (0.65-2.18) | 1.23 (0.64-2.39) | 45 (14) | 0.95 (0.53-1.68) | 0.80 (0.44-1.47) |
| **VEGETABLES** |  |  |  |  |  |  |  |  |  |  |
| Broccoli | *Overweight/Obese* | 671 (70) |  |  | 347 (70) |  |  | 324 (70) |  |  |
|  | Never | 583 (87) | 1.00 (Ref) | 1.00 (Ref) | 310 (89) | 1.00 (Ref) | 1.00 (Ref) | 273 (84) | 1.00 (Ref) | 1.00 (Ref) |
|  | 1-3 days | 53 (8) | 0.98 (0.59-1.62) | 0.89 (0.53-1.49) | 20 (6) | 0.61 (0.30-1.24) | 0.53 (0.25-1.02) | 33 (10) | 1.52 (0.73-1.19) | 1.47 (0.68-3.20) |
|  | 4-7 days | 35 (5) | 1.55 (0.76-3.18) | 1.42 (0.68-2.96) | 17 (5) | 1.81 (0.60-5.89) | 1.74 (0.56-5.48) | 18 (6) | 1.38 (0.54-3.58) | 1.24 (0.47-3.26) |
|  |  |  |  |  |  |  |  |  |  |  |
| Spinach (including morogo) | *Overweight/Obese* | 671 (70) |  |  | 347 (70) |  |  | 324 (70) |  |  |
|  | Never | 338 (50) | 1.00 (Ref) | 1.00 (Ref) | 183 (53) | 1.00 (Ref) | 1.00 (Ref) | 155 (48) | 1.00 (Ref) | 1.00 (Ref) |
|  | 1-3 days | 224 (33) | 1.05 (0.77-1.42) | 0.89 (0.53-1.49) | 106 (31) | 1.13 (0.73-1.75) | 0.94 (0.59-1.49) | 118 (36) | 0.96 (0.62-1.48) | 0.86 (0.54-1.35) |
|  | 4-7 days | 109 (16) | 1.03 (0.70-1.52) | 0.85 (0.56-1.30) | 58 (17) | 1.36 (0.77-2.41) | 1.08 (0.59-1.99) | 51 (16) | 0.78 (0.45-1.35) | 0.68 (0.38-1.24) |
|  |  |  |  |  |  |  |  |  |  |  |
| Carrots | *Overweight/Obese* | 671 (70) |  |  | 347 (70) |  |  | 324 (70) |  |  |
|  | Never | 153 (23) | 1.00 (Ref) | 1.00 (Ref) | 77 (22) | 1.00 (Ref) | 1.00 (Ref) | 76 (23) | 1.00 (Ref) | 1.00 (Ref) |
|  | 1-3 days | 281 (42) | 1.09 (0.76-1.55) | 1.01 (0.70-1.46) | 141 (41) | 1.47 (0.91-2.38) | 1.37 (0.83-2.26) | 140 (43) | 0.74 (0.43-1.28) | 0.70 (0.40-1.24) |
|  | 4-7 days | 237 (35) | 1.04 (0.72-1.50) | 0.97 (0.67-1.42) | 129 (37) | 1.54 (0.94-2.52) | 1.40 (0.84-2.33) | 108 (33) | 0.65 (0.37-1.23) | 0.62 (0.34-1.10) |
|  |  |  |  |  |  |  |  |  |  |  |
| Tomato (raw/cooked) | *Overweight/Obese* | 671 (70) |  |  | 347 (70) |  |  | 324 (70) |  |  |
|  | Never | 270 (40) | 1.00 (Ref) | 1.00 (Ref) | 137 (39) | 1.00 (Ref) | 1.00 (Ref) | 133 (41) | 1.00 (Ref) | 1.00 (Ref) |
|  | 1-3 days | 210 (31) | 0.83 (0.60-1.16) | 0.80 (0.57-1.12) | 104 (30) | 0.88 (0.56-1.37) | 0.89 (0.56-1.41) | 106 (33) | 0.78 (0.48-1.26) | 0.72 (0.43-1.19) |
|  | 4-7 days | 191 (28) | 0.86 (0.61-1.21) | 0.75 (0.52-1.07) | 106 (31) | 1.22 (0.76-1.97) | 1.10 (0.66-1.82) | 85 (26) | **0.60 (0.37-0.98)** | **0.50 (0.30-0.84)** |
|  |  |  |  |  |  |  |  |  |  |  |
| Green beans | *Overweight/Obese* | 671 (70) |  |  | 347 (70) |  |  | 324 (70) |  |  |
|  | Never | 472 (70) | 1.00 (Ref) | 1.00 (Ref) | 257 (74) | 1.00 (Ref) | 1.00 (Ref) | 215 (66) | 1.00 (Ref) | 1.00 (Ref) |
|  | 1-3 days | 160 (24) | 1.15 (0.82-1.62) | 1.01 (0.71-1.42) | 71 (20) | 0.98 (0.61-1.58) | 1.92 (0.56-1.49) | 89 (27) | 1.34 (0.83-2.18) | 1.15 (0.70-1.89) |
|  | 4-7 days | 39 (6) | 0.60 (0.36-1.01) | **0.52 (0.30-0.90)** | 19 (53) | 0.90 (0.40-2.06) | 0.76 (0.33-1.72) | 20 (6) | **0.46 (0.23-0.90)** | **0.41 (0.20-0.86)** |
|  |  |  |  |  |  |  |  |  |  |  |
| Green peas | *Overweight/Obese* | 671 (70) |  |  | 347 (70) |  |  | 324 (70) |  |  |
|  | Never | 448 (67) | 1.00 (Ref) | 1.00 (Ref) | 239 (69) | 1.00 (Ref) | 1.00 (Ref) | 209 (65) | 1.00 (Ref) | 1.00 (Ref) |
|  | 1-3 days | 173 (26) | **1.43 (1.02-2.02)** | 1.30 (0.91-1.85) | 82 (24) | 1.15 (0.72-1.83) | 1.04 (0.64-1.68) | 91 (28) | **1.83 (1.10-3.05)** | 1.69 (1.01-2.87) |
|  | 4-7 days | 50 (7) | 0.83 (0.51-1.36) | 0.72 (0.42-1.22) | 26 (7) | 1.06 (0.50-2.22) | 0.88 (0.40-1.93) | 24 (7) | 0.68 (0.35-1.33) | 0.62 (0.30-1.27) |
|  |  |  |  |  |  |  |  |  |  |  |
| Mixed vegetables | *Overweight/Obese* | 671 (70) |  |  | 347 (70) |  |  | 324 (70) |  |  |
|  | Never | 298 (44) | 1.00 (Ref) | 1.00 (Ref) | 149 (43) | 1.00 (Ref) | 1.00 (Ref) | 149 (46) | 1.00 (Ref) | 1.00 (Ref) |
|  | 1-3 days | 237 (35) | 1.23 (0.90-1.70) | 1.06 (0.76-1.48) | 119 (34) | 1.28 (0.83-2.00) | 1.12 (0.70-1.80) | 118 (36) | 1.18 (0.74-1.87) | 0.98 (0.60-1.59) |
|  | 4-7 days | 136 (20) | 0.88 (0.62-1.25) | 0.79 (0.55-1.14) | 79 (23) | 1.31 (0.79-2.17) | 1.21 (0.72-2.02) | 57 (18) | **0.58 (0.35-0.96)** | **0.49 (0.29-0.84)** |
|  |  |  |  |  |  |  |  |  |  |  |
| Pumpkin/butternut | *Overweight/Obese* | 671 (70) |  |  | 347 (70) |  |  | 324 (70) |  |  |
|  | Never | 250 (37) | 1.00 (Ref) | 1.00 (Ref) | 131 (38) | 1.00 (Ref) | 1.00 (Ref) | 119 (37) | 1.00 (Ref) | 1.00 (Ref) |
|  | 1-3 days | 221 (33) | **1.60 (1.15-2.24)** | **1.62 (1.15-2.29)** | 115 (33) | **2.06 (1.27-3.34)** | **2.13 (1.29-3.49)** | 106 (33) | 1.26 (0.79-2.02) | 1.29 (0.79-2.10) |
|  | 4-7 days | 199 (30) | 1.34 (0.96-1.86) | 1.31 (0.93-1.85) | 100 (29) | 1.33 (0.84-2.10) | 1.34 (0.83-2.17) | 99 (31) | 1.33 (0.82-2.17) | 1.26 (0.76-2.09) |
| **LEGUMES** |  |  |  |  |  |  |  |  |  |  |
| Legumes e.g. baked beans, lentils | *Overweight/Obese* | 671 (70) |  |  | 347 (70) |  |  | 324 (70) |  |  |
|  | Never | 419 (62) | 1.00 (Ref) | 1.00 (Ref) | 216 (62) | 1.00 (Ref) | 1.00 (Ref) | 203 (63) | 1.00 (Ref) | 1.00 (Ref) |
|  | 1-3 days | 162 (24) | 0.99 (0.71-1.38) | 0.90 (0.64-1.27) | 84 (24) | 1.00 (0.64-1.58) | 0.89 (0.55-1.45) | 78 (24) | 0.97 (0.60-1.58) | 0.89 (0.54-1.48) |
|  | 4-7 days | 90 (13) | 0.89 (0.60-1.34) | 0.79 (0.52-1.20) | 47 (14) | 1.54 (0.80-2.90) | 1.43 (0.75-2.73) | 43 (13) | 0.59 (0.35-1.01) | **0.50 (0.28-0.86)** |
|  |  |  |  |  |  |  |  |  |  |  |
| Peanut and nuts | *Overweight/Obese* | 671 (70) |  |  | 347 (70) |  |  | 324 (70) |  |  |
|  | Never | 512 (76) | 1.00 (Ref) | 1.00 (Ref) | 269 (78) | 1.00 (Ref) | 1.00 (Ref) | 243 (75) | 1.00 (Ref) | 1.00 (Ref) |
|  | 1-3 days | 95 (14) | 0.97 (0.65-1.44) | 0.93 (0.62-1.39) | 46 (13) | 0.82 (0.47-1.41) | 0.77 (0.44-1.35) | 49 (15) | 1.17 (0.66-2.08) | 1.13 (0.63-2.03) |
|  | 4-7 days | 64 (10) | 0.92 (0.58-1.45) | 0.86 (0.53-1.39) | 32 (9) | 0.77 (0.41-1.44) | 0.77 (0.39-1.51) | 32 (10) | 1.11 (0.56-2.21) | 0.97 (0.47-1.98) |
|  |  |  |  |  |  |  |  |  |  |  |
| **Fats/oils** | *Overweight/Obese* | 671 (70) |  |  | 347 (70) |  |  | 324 (70) |  |  |
| Soft margarine (tub) | Never | 225 (34) | 1.00 (Ref) | 1.00 (Ref) | 119 (34) | 1.00 (Ref) | 1.00 (Ref) | 106 (33) | 1.00 (Ref) | 1.00 (Ref) |
|  | 1-3 days | 182 (27) | 1.01 (0.71-1.45) | 0.91 (0.63-1.30) | 86 (25) | 0.72 (0.45-1.17) | 0.62 (0.37-1.04) | 96 (30) | 1.51 (0.89-2.57) | 1.38 (0.80-2.38) |
|  | 4-7 days | 264 (39) | 1.02 (0.74-1.41) | 0.92 (0.65-1.29) | 142 (41) | 1.12 (0.71-1.78) | 0.98 (0.60-1.60) | 122 (38) | 0.93 (0.59-1.46) | 0.85 (0.53-1.38) |

Adjusted model included maternal age, socio-economic status, relationship status, alcohol use and parity. Missing data n (%): Socio-economic status 2 (0.2), relationship status 5 (.05), alcohol use 1 (0.1). Where data are missing on predictors, cases were included in the reference category in the regression. Interpretation of OR’s: consumption of ‘food group’ for ‘frequency’ a week was associated with increased (OR>1) or decreases (OR<1) odds of overweight/obesity compared to not consuming ‘food group’ in the past 7 days.
