# Supplementary material for: Association between food intake and obesity in pregnant women living with and without HIV in Cape Town, South Africa: a prospective cohort study
Source: BMC Public Health. 2021 Aug 4;21:1504. doi: 10.1186/s12889-021-11566-2 (PMC8335890; doi:10.1186/s12889-021-11566-2)
Supplement: Supplementary file 4 — Additional file 4. Association between food consumption frequency and maternal GWG, overall and stratified by HIV status. [file 12889_2021_11566_MOESM4_ESM.docx]

| Additional file 3. Association between food consumption frequency and maternal GWG, overall and stratified by HIV status |
| --- |

|  |  |  | | | **HIV status** | | | | | |
| --- | --- | --- | --- | --- | --- | --- | --- | --- | --- | --- |
|  |  | **Overall** | | | **Without HIV** | | | **With HIV** | | |
|  |  |  | **Unadjusted** | **Adjusted** |  | **Unadjusted** | **Adjusted** |  | **Unadjusted** | **Adjusted** |
| **Food group** | **GWG (kg/week)** | **Total**  **N (%) = 746** | **OR (95% CI)** | **aOR (95% CI)** | **Total**  **N (%) = 363** | **OR (95% CI)** | **aOR (95% CI)** | **Total**  **N (%) = 383** | **OR (95% CI)** | **aOR (95% CI)** |
| **STARCH**  Brown/whole wheat bread/rolls |  |  |  |  |  |  |  |  |  |  |
|  | *Excessive* | 361 (48) |  |  | 199 (55) |  |  | 162 (42) |  |  |
|  | Never | 40 (11) | 1.00 (Ref) | 1.00 (Ref) | 25 (13) | 1.00 (Ref) | 1.00 (Ref) | 15 (9) | 1.00 (Ref) | 1.00 (Ref) |
|  | 1-3 days | 105 (29) | 1.41 (0.68-2.91) | 1.40 (0.68-2.88) | 65 (33) | 1.40 (0.50-3.92) | 1.31 (0.47-3.66) | 40 (25) | 1.42 (0.50-4.04) | 1.50 (0.51-4.42) |
|  | 4-7 days | 216 (60) | 1.04 (0.34-1.98) | 1.05 (0.54-2.01) | 109 (55) | 0.90 (0.36-2.26) | 0.85 (0.34-2.12) | 107 (66) | 1.30 (0.51-3.28) | 1.43 (0.54-3.78) |
| Breakfast cereal (instant) |  |  |  |  |  |  |  |  |  |  |
|  | *Excessive* | 361 (48) |  |  | 199 (55) |  |  | 162 (42) |  |  |
|  | Never | 189 (52) | 1.00 (Ref) | 1.00 (Ref) | 102 (51) | 1.00 (Ref) | 1.00 (Ref) | 87 (54) | 1.00 (Ref) | 1.00 (Ref) |
|  | 1-3 days | 71 (20) | 1.00 (0.57-1.73) | 1.00 (0.57-1.75) | 41 (21) | 1.21 (0.64-3.78) | 1.18 (0.51-2.73) | 30 (19) | 0.84 (0.40-1.77) | 0.86 (0.39-1.87) |
|  | 4-7 days | 101 (28) | 0.88 (0.55-1.42) | 0.90 (0.56-1.45) | 56 (28) | 0.89 (0.42-1.88) | 0.84 (0.42-1.66) | 45 (28) | 0.93 (0.47-1.80) | 1.01 (0.51-1.99) |
| Oats porridge |  |  |  |  |  |  |  |  |  |  |
|  | *Excessive* | 361 (48) |  |  | 199 (55) |  |  | 162 (42) |  |  |
|  | Never | 222 (63) | 1.00 (Ref) | 1.00 (Ref) | 125 (63) | 1.00 (Ref) | 1.00 (Ref) | 104 (64) | 1.00 (Ref) | 1.00 (Ref) |
|  | 1-3 days | 67 (19) | 1.27 (0.71-2.27) | 1.29 (0.71-2.32) | 38 (19) | 1.33 (0.57-3.11) | 1.49 (0.63-3.55) | 29 (18) | 1.20 (0.54-2.68) | 1.23 (0.53-2.82) |
|  | 4-7 days | 65 (18) | 0.89 (0.52-1.50) | 0.91 (0.54-1.54) | 36 (18) | 0.92 (0.42-1.99) | 0.94 (0.43-2.06) | 29 (18) | 0.86 (0.41-1.78) | 0.85 (0.40-1.78) |
| Sweet potato |  |  |  |  |  |  |  |  |  |  |
|  | *Excessive* | 361 (48) |  |  | 199 (55) |  |  | 162 (42) |  |  |
|  | Never | 282 (78) | 1.00 (Ref) | 1.00 (Ref) | 152 (76) | 1.00 (Ref) | 1.00 (Ref) | 130 (80) | 1.00 (Ref) | 1.00 (Ref) |
|  | 1-3 days | 42 (12) | 1.25 (0.63-2.48) | 1.25 (0.63-2.49) | 27 (14) | 1.63 (0.59-4.49) | 1.77 (0.64-4.93) | 15 (9) | 0.91 (0.35-2.35) | 0.81 (0.31-2.14) |
|  | 4-7 days | 37 (10) | 1.65 (0.75-3.68) | 1.77 (0.79-3.94) | 20 (10) | 2.02 (0.57-7.11) | 2.18 (0.61-7.73) | 17 (10) | 1.44 (0.50-4.10) | 1.53 (0.52-4.46) |
| Potato (any preparation) |  |  |  |  |  |  |  |  |  |  |
|  | *Excessive* | 361 (48) |  |  | 199 (55) |  |  | 162 (42) |  |  |
|  | Never | 38 (11) | 1.00 (Ref) | 1.00 (Ref) | 21 (11) | 1.00 (Ref) | 1.00 (Ref) | 17 (10) | 1.00 (Ref) | 1.00 (Ref) |
|  | 1-3 days | 108 (30) | 1.24 (0.63-2.44) | 1.15 (0.57-2.30) | 59 (30) | 1.40 (0.52-3.76) | 1.31 (0.49-3.51) | 49 (30) | 1.13 (0.44-2.91) | 1.02 (0.38-2.74) |
|  | 4-7 days | 215 (60) | 1.48 (0.78-2.80) | 1.42 (0.74-2.71) | 119 (60) | 1.51 (0.61-3.75) | 1.44 (0.58-3.56) | 96 (59) | 1.45 (0.59-3.56) | 1.38 (0.54-3.52) |
| **PROTEIN** |  |  |  |  |  |  |  |  |  |  |
| Red meat (any) | *Excessive* | 361 (48) |  |  | 199 (55) |  |  | 162 (42) |  |  |
|  | Never | 173 (48) | 1.00 (Ref) | 1.00 (Ref) | 92 (46) | 1.00 (Ref) | 1.00 (Ref) | 81 (50) | 1.00 (Ref) | 1.00 (Ref) |
|  | 1-3 days | 124 (34) | 1.43 (0.89-2.31) | 1.38 (0.85-2.23) | 75 (38) | 1.53 (0.77-3.02) | 1.51 (0.77-3.00) | 49 (30) | 1.28 (0.65-2.52) | 1.23 (0.62-2.44) |
|  | 4-7 days | 64 (18) | 1.11 (0.63-1.95) | 1.08 (0.62-1.90) | 32 (16) | 1.30 (0.54-3.14) | 1.29 (0.53-3.10) | 32 (20) | 1.02 (0.48-2.13) | 0.97 (0.46-2.04) |
|  |  |  |  |  |  |  |  |  |  |  |
| Organ meat e.g. liver | *Excessive* | 361 (48) |  |  | 199 (55) |  |  | 162 (42) |  |  |
|  | Never | 244 (68) | 1.00 (Ref) | 1.00 (Ref) | 131 (66) | 1.00 (Ref) | 1.00 (Ref) | 113 (70) | 1.00 (Ref) | 1.00 (Ref) |
|  | 1-3 days | 75 (21) | 1.03 (0.60-1.75) | 1.01 (0.59-1.73) | 48 (24) | 1.42 (0.64-3.19) | 1.45 (0.64-3.25) | 27 (17) | 1.72 (0.34-1.50) | 0.64 (0.30-1.36) |
|  | 4-7 days | 42 (12) | 0.63 (0.35-1.13) | 0.63 (0.35-1.14) | 20 (10) | 0.53 (0.23-1.25) | 0.55 (0.24-1.28) | 22 (14) | 0.74 (0.33-1.67) | 0.76 (0.34-1.73) |
|  |  |  |  |  |  |  |  |  |  |  |
| Chicken (any) | *Excessive* | 361 (48) |  |  | 199 (55) |  |  | 162 (42) |  |  |
|  | Never | 51 (14) | 1.00 (Ref) | 1.00 (Ref) | 32 (16) | 1.00 (Ref) | 1.00 (Ref) | 19 (12) | 1.00 (Ref) | 1.00 (Ref) |
|  | 1-3 days | 116 (32) | 1.11 (0.59-2.08) | 1.04 (0.55-1.98) | 61 (31) | 1.00 (0.43-2.33) | 0.92 (0.39-2.20) | 55 (34) | 1.30 (0.51-3.35) | 1.19 (0.46-3.04) |
|  | 4-7 days | 194 (54) | 1.27 (0.70-2.29) | 1.24 (0.68-2.25) | 106 (53) | 1.66 (0.73-3.78) | 1.62 (0.71-3.71) | 88 (54) | 1.10 (0.45-2.65) | 1.06 (0.44-2.57) |
|  |  |  |  |  |  |  |  |  |  |  |
| Tinned fish | *Excessive* | 361 (48) |  |  | 199 (55) |  |  | 162 (42) |  |  |
|  | Never | 238 (66) | 1.00 (Ref) | 1.00 (Ref) | 139 (70) | 1.00 (Ref) | 1.00 (Ref) | 99 (61) | 1.00 (Ref) | 1.00 (Ref) |
|  | 1-3 days | 74 (21) | 0.90 (0.54-1.50) | 0.91 (0.55-1.52) | 38 (19) | 0.82 (0.39-1.73) | 0.81 (0.38-1.71) | 36 (22) | 1.02 (0.50-2.06) | 0.99 (0.49-2.00) |
|  | 4-7 days | 49 (14) | 1.00 (0.54-1.87) | 1.02 (0.55-1.89) | 22 (11) | 0.95 (0.36-2.52) | 1.01 (0.37-2.77) | 27 (17) | 1.15 (0.51-2.58) | 1.07 (0.47-2.42) |
|  |  |  |  |  |  |  |  |  |  |  |
| Eggs (any) | *Excessive* | 361 (48) |  |  | 199 (55) |  |  | 162 (42) |  |  |
|  | Never | 120 (33) | 1.00 (Ref) | 1.00 (Ref) | 70 (35) | 1.00 (Ref) | 1.00 (Ref) | 50 (31) | 1.00 (Ref) | 1.00 (Ref) |
|  | 1-3 days | 136 (38) | 1.11 (0.67-1.82) | 1.11 (0.67-1.85) | 65 (31) | 0.93 (0.43-2.01) | 0.96 (0.44-2.09) | 71 (44) | 1.36 (0.70-2.66) | 1.46 (0.73-2.90) |
|  | 4-7 days | 105 (29) | 0.88 (0.52-1.46) | 0.88 (0.53-1.47) | 64 (32) | 0.66 (0.32-1.38) | 0.68 (0.33-1.41) | 41 (25) | 1.09 (0.52-2.29) | 1.16 (0.56-2.43) |
| **DAIRY** |  |  |  |  |  |  |  |  |  |  |
| Milk/yoghurt/maas to drink/on cereals | *Excessive* | 361 (48) |  |  | 199 (55) |  |  | 162 (42) |  |  |
|  | Never | 58 (16) | 1.00 (Ref) | 1.00 (Ref) | 30 (15) | 1.00 (Ref) | 1.00 (Ref) | 28 (17) | 1.00 (Ref) | 1.00 (Ref) |
|  | 1-3 days | 129 (36) | 1.20 (0.64-2.25) | 1.15 (0.61-2.16) | 71 (36) | 1.78 (0.75-4.21) | 1.62 (0.69-3.84) | 58 (36) | 0.79 (0.31-2.00) | 0.77 (0.30-1.99) |
|  | 4-7 days | 174 (48) | 0.94 (0.39-1.31) | 0.96 (0.53-1.73) | 98 (49) | 1.51 (0.68-3.35) | 1.46 (0.66-3.23) | 76 (47) | 0.57 (0.24-1.38) | 0.62 (0.25-1.51) |
|  |  |  |  |  |  |  |  |  |  |  |
| Milk in tea/coffee | *Excessive* | 361 (48) |  |  | 199 (55) |  |  | 162 (42) |  |  |
|  | Never | 161 (45) | 1.00 (Ref) | 1.00 (Ref) | 97 (49) | 1.00 (Ref) | 1.00 (Ref) | 64 (40) | 1.00 (Ref) | 1.00 (Ref) |
|  | 1-3 days | 73 (20) | 1.67 (0.90-3.10) | 1.64 (0.88-3.06) | 40 (20) | **6.19 (1.41-27.17)** | **6.04 (1.37-26.50)** | 33 (20) | 1.07 (0.50-2.29) | 1.05 (0.48-2.30) |
|  | 4-7 days | 227 (35) | 1.01 (0.64-1.59) | 1.03 (0.65-1.63) | 62 (31) | 0.87 (0.46-1.65) | 0.86 (0.45-1.66) | 65 (40) | 1.23 (0.65-2.33) | 1.26 (0.66-2.42) |
|  |  |  |  |  |  |  |  |  |  |  |
| Cheese (except cottage) | *Excessive* | 361 (48) |  |  | 199 (55) |  |  | 162 (42) |  |  |
|  | Never | 219 (61) | 1.00 (Ref) | 1.00 (Ref) | 129 (65) | 1.00 (Ref) | 1.00 (Ref) | 90 (56) | 1.00 (Ref) | 1.00 (Ref) |
|  | 1-3 days | 83 (21) | 0.71 (0.44-1.16) | 0.67 (0.41-1.09) | 42 (21) | 0.70 (0.34-1.44) | 0.66 (0.31-1.38) | 41 (25) | 0.78 (0.41-1.50) | 0.75 (0.38-1.48) |
|  | 4-7 days | 59 (16) | 0.89 (0.50-1.58) | 0.83 (0.46-1.50) | 28 (14) | 0.65 (0.29-1.49) | 0.58 (0.25-1.33) | 31 (19) | 1.24 (0.55-2.79) | 1.26 (0.54-2.95) |
| **FRUITS** |  |  |  |  |  |  |  |  |  |  |
| Citrus fruit e.g. orange | *Excessive* | 361 (48) |  |  | 199 (55) |  |  | 162 (42) |  |  |
|  | Never | 190 (53) | 1.00 (Ref) | 1.00 (Ref) | 105 (53) | 1.00 (Ref) | 1.00 (Ref) | 85 (52) | 1.00 (Ref) | 1.00 (Ref) |
|  | 1-3 days | 102 (28) | 0.89 (0.55-1.47) | 0.88 (0.53-1.45) | 62 (31) | 1.10 (0.53-2.26) | 1.09 (0.53-2.27) | 40 (25) | 0.72 (0.36-1.43) | 0.68 (0.34-1.36) |
|  | 4-7 days | 68 (19) | 0.60 (0.36-1.00) | 0.60 (0.36-1.00) | 32 (16) | 0.57 (0.26-1.21) | 0.59 (0.27-1.28) | 36 (22) | 0.65 (0.32-1.30) | 0.67 (0.33-1.37) |
|  |  |  |  |  |  |  |  |  |  |  |
| Pure orange/guava juice | *Excessive* | 361 (48) |  |  | 199 (55) |  |  | 162 (42) |  |  |
|  | Never | 217 (60) | 1.00 (Ref) | 1.00 (Ref) | 119 (60) | 1.00 (Ref) | 1.00 (Ref) | 98 (60) | 1.00 (Ref) | 1.00 (Ref) |
|  | 1-3 days | 92 (25) | 0.89 (0.54-1.46) | 0.88 (0.53-1.44) | 51 (26) | 0.92 (0.44-1.93) | 0.92 (0.44-1.92) | 41 (25) | 0.86 (0.44-1.68) | 0.82 (0.41-1.63) |
|  | 4-7 days | 52 (14) | 0.62 (0.36-1.08) | 0.63 (0.37-1.10) | 29 (15) | 0.52 (0.24-1.14) | 0.55 (0.25-1.18) | 23 (14) | 0.73 (0.33-1.60) | 0.72 (0.32-1.58) |
|  |  |  |  |  |  |  |  |  |  |  |
| Banana | *Excessive* | 361 (48) |  |  | 199 (55) |  |  | 162 (42) |  |  |
|  | Never | 89 (25) | 1.00 (Ref) | 1.00 (Ref) | 48 (24) | 1.00 (Ref) | 1.00 (Ref) | 41 (25) | 1.00 (Ref) | 1.00 (Ref) |
|  | 1-3 days | 146 (40) | 1.34 (0.80-2.24) | 1.34 (0.80-2.23) | 77 (39) | 1.69 (0.81-3.55) | 1.67 (0.80-3.50) | 69 (43) | 1.10 (0.53-2.27) | 1.13 (0.55-2.34) |
|  | 4-7 days | 126 (35) | 1.24 (0.74-2.10) | 1.27 (0.75-2.15) | 74 (37) | 1.72 (0.81-3.65) | 1.71 (0.81-3.62) | 52 (32) | 0.90 (0.43-1.89) | 0.94 (0.44-2.02) |
|  |  |  |  |  |  |  |  |  |  |  |
| Mangoes | *Excessive* | 361 (48) |  |  | 199 (55) |  |  | 162 (42) |  |  |
|  | Never | 326 (90) | 1.00 (Ref) | 1.00 (Ref) | 182 (91) | 1.00 (Ref) | 1.00 (Ref) | 144 (89) | 1.00 (Ref) | 1.00 (Ref) |
|  | 1-3 days | 24 (7) | 0.70 (0.33-1.48) | 0.69 (0.33-1.46) | 12 (6) | 0.49 (0.18-1.39) | 0.51 (0.18-1.44) | 12 (7) | 1.00 (0.34-2.97) | 0.90 (0.30-2.68) |
|  | 4-7 days | 11 (3) | 0.71 (0.24-2.09) | 0.70 (0.24-2.07) | 5 (3) | 0.41 (0.09-1.79) | 0.41 (0.09-1.87) | 6 (4) | 1.25 (0.24-6.38) | 1.19 (0.23-6.27) |
|  |  |  |  |  |  |  |  |  |  |  |
| Apples/pears | *Excessive* | 361 (48) |  |  | 199 (55) |  |  | 162 (42) |  |  |
|  | Never | 79 (22) | 1.00 (Ref) | 1.00 (Ref) | 38 (19) | 1.00 (Ref) | 1.00 (Ref) | 41 (25) | 1.00 (Ref) | 1.00 (Ref) |
|  | 1-3 days | 148 (41) | 1.05 (0.61-1.82) | 1.06 (0.61-1.84) | 83 (42) | 1.61 (0.73-3.55) | 1.63 (0.73-3.63) | 65 (40) | 0.71 (0.33-1.52) | 0.69 (0.32-1.51) |
|  | 4-7 days | 134 (37) | 1.00 (0.57-1.73) | 1.03 (0.59-1.72) | 78 (39) | 1.37 (0.63-2.99) | 1.42 (0.65-3.12) | 56 (35) | 0.71 (0.32-1.55) | 0.74 (0.34-1.63) |
|  |  |  |  |  |  |  |  |  |  |  |
| Avocado | *Excessive* | 361 (48) |  |  | 199 (55) |  |  | 162 (42) |  |  |
|  | Never | 259 (72) | 1.00 (Ref) | 1.00 (Ref) | 145 (73) | 1.00 (Ref) | 1.00 (Ref) | 114 (70) | 1.00 (Ref) | 1.00 (Ref) |
|  | 1-3 days | 55 (15) | 0.67 (0.39-1.16) | 0.63 (0.36-1.10) | 31 (16) | 0.68 (0.31-1.49) | 0.59 (0.26-1.35) | 24 (15) | 0.66 (0.31-1.43) | 0.66 (0.30-1.43) |
|  | 4-7 days | 47 (13) | 0.66 (0.37-1.17) | 0.64 (0.36-1.16) | 23 (12) | 0.69 (0.29-1.68) | 0.69 (0.28-1.69) | 24 (15) | 0.66 (0.31-1.43) | 0.67 (0.30-1.50) |
| **VEGETABLES** |  |  |  |  |  |  |  |  |  |  |
| Broccoli | *Excessive* | 361 (48) |  |  | 199 (55) |  |  | 162 (42) |  |  |
|  | Never | 319 (88) | 1.00 (Ref) | 1.00 (Ref) | 181 (91) | 1.00 (Ref) | 1.00 (Ref) | 138 (85) | 1.00 (Ref) | 1.00 (Ref) |
|  | 1-3 days | 27 (7) | 0.73 (0.36-1.50) | 0.69 (0.33-1.42) | 11 (6) | 0.71 (0.23-2.35) | 0.67 (0.20-2.30) | 16 (10) | 0.83 (0.33-2.04) | 0.79 (0.31-1.99) |
|  | 4-7 days | 15 (4) | 0.98 (0.35-2.76) | 1.92 (0.34-2.59) | 7 (4) | 0.61 (0.15-2.44) | 0.61 (0.15-2.47) | 8 (5) | 1.65 (0.34-8.04) | 1.57 (0.33-7.37) |
|  |  |  |  |  |  |  |  |  |  |  |
| Spinach (including morogo) | *Excessive* | 361 (48) |  |  | 199 (55) |  |  | 162 (42) |  |  |
|  | Never | 202 (56) | 1.00 (Ref) | 1.00 (Ref) | 118 (59) | 1.00 (Ref) | 1.00 (Ref) | 84 (52) | 1.00 (Ref) | 1.00 (Ref) |
|  | 1-3 days | 98 (27) | 0.67 (0.42-1.07) | 0.66 (0.41-1.07) | 46 (23) | 0.55 (0.28-1.09) | 0.56 (0.28-1.13) | 52 (32) | 0.83 (0.44-1.58) | 0.81 (0.42-1.58) |
|  | 4-7 days | 61 (17) | 0.83 (0.47-1.48) | 0.84 (0.47-1.51) | 35 (18) | 1.00 (0.42-2.40) | 1.00 (0.41-2.45) | 26 (16) | 0.74 (0.34-1.62) | 0.75 (0.31-1.64) |
|  |  |  |  |  |  |  |  |  |  |  |
| Carrots | *Excessive* | 361 (48) |  |  | 199 (55) |  |  | 162 (42) |  |  |
|  | Never | 84 (23) | 1.00 (Ref) | 1.00 (Ref) | 50 (25) | 1.00 (Ref) | 1.00 (Ref) | 34 (21) | 1.00 (Ref) | 1.00 (Ref) |
|  | 1-3 days | 143 (40) | 1.06 (0.63-1.78) | 1.04 (0.61-1.75) | 78 (39) | 1.95 (0.92-4.12) | 1.94 (0.91-4.11) | 65 (40) | 0.62 (0.28-1.37) | 0.58 (0.26-1.32) |
|  | 4-7 days | 134 (37) | 1.24 (0.72-2.13) | 1.26 (0.72-2.18) | 71 (36) | 1.58 (0.76-3.29) | 1.69 (0.80-3.58) | 63 (39) | 0.93 (0.40-2.14) | 0.92 (0.39-2.16) |
|  |  |  |  |  |  |  |  |  |  |  |
| Tomato (raw/cooked) | *Excessive* | 361 (48) |  |  | 199 (55) |  |  | 162 (42) |  |  |
|  | Never | 148 (41) | 1.00 (Ref) | 1.00 (Ref) | 75 (38) | 1.00 (Ref) | 1.00 (Ref) | 73 (45) | 1.00 (Ref) | 1.00 (Ref) |
|  | 1-3 days | 111 (31) | 1.16 (0.69-1.94) | 1.14 (0.68-1.91) | 60 (30) | 1.48 (0.69-3.15) | 1.45 (0.68-3.09) | 51 (31) | 0.93 (0.46-1.89) | 0.92 (0.45-1.89) |
|  | 4-7 days | 102 (28) | 0.79 (0.48-1.28) | 0.77 (0.47-1.26) | 64 (32) | 1.20 (0.59-2.44) | 1.23 (0.60-2.53) | 38 (23) | **0.50 (0.25-0.99)** | **0.48 (0.24-0.96)** |
|  |  |  |  |  |  |  |  |  |  |  |
| Green beans | *Excessive* | 361 (48) |  |  | 199 (55) |  |  | 162 (42) |  |  |
|  | Never | 272 (75) | 1.00 (Ref) | 1.00 (Ref) | 152 (76) | 1.00 (Ref) | 1.00 (Ref) | 120 (74) | 1.00 (Ref) | 1.00 (Ref) |
|  | 1-3 days | 69 (19) | 0.86 (0.51-1.45) | 1.84 (0.50-1.41) | 39 (20) | 1.88 (0.75-4.74) | 1.85 (0.74-4.64) | 30 (19) | 0.54 (0.27-1.06) | 0.53 (0.27-1.05) |
|  | 4-7 days | 20 (6) | 0.57 (0.26-1.23) | 0.55 (0.24-1.22) | 8 (4) | 0.58 (0.17-2.02) | 0.65 (0.14-2.10) | 12 (7) | 0.59 (0.22-1.59) | 0.59 (0.21-1.65) |
|  |  |  |  |  |  |  |  |  |  |  |
| Green peas | *Excessive* | 361 (48) |  |  | 199 (55) |  |  | 162 (42) |  |  |
|  | Never | 262 (73) | 1.00 (Ref) | 1.00 (Ref) | 147 (74) | 1.00 (Ref) | 1.00 (Ref) | 115 (71) | 1.00 (Ref) | 1.00 (Ref) |
|  | 1-3 days | 72 (20) | 0.68 (0.42-1.12) | 0.67 (0.41-1.10) | 41 (21) | 0.68 (0.33-1.38) | 0.67 (0.33-1.35) | 31 (19) | 0.68 (0.34-1.36) | 0.67 (0.33-1.34) |
|  | 4-7 days | 27 (7) | 0.61 (0.30-1.24) | 0.58 (0.28-1.20) | 11 (6) | 0.43 (0.15-1.23) | 0.78 (0.13-1.14) | 16 (10) | 0.85 (0.33-2.22) | 0.81 (0.30-2.20) |
|  |  |  |  |  |  |  |  |  |  |  |
| Mixed vegetables | *Excessive* | 361 (48) |  |  | 199 (55) |  |  | 162 (42) |  |  |
|  | Never | 177 (49) | 1.00 (Ref) | 1.00 (Ref) | 90 (45) | 1.00 (Ref) | 1.00 (Ref) | 87 (54) | 1.00 (Ref) | 1.00 (Ref) |
|  | 1-3 days | 117 (32) | 1.17 (0.71-1.93) | 1.13 (0.67-1.67) | 68 (34) | 1.46 (0.71-2.99) | 1.44 (0.70-2.97) | 49 (30) | 0.93 (0.46-1.86) | 0.90 (0.45-1.84) |
|  | 4-7 days | 67 (19) | **0.59 (0.36-0.99)** | 0.58 (0.35-0.98) | 41 (21) | 0.95 (0.44-2.02) | 0.94 (0.44-2.03) | 26 (16) | **0.38 (0.19-0.77)** | **0.38 (0.18-0.78)** |
|  |  |  |  |  |  |  |  |  |  |  |
| Pumpkin/butternut | *Excessive* | 361 (48) |  |  | 199 (55) |  |  | 162 (42) |  |  |
|  | Never | 151 (42) | 1.00 (Ref) | 1.00 (Ref) | 89 (45) | 1.00 (Ref) | 1.00 (Ref) | 62 (38) | 1.00 (Ref) | 1.00 (Ref) |
|  | 1-3 days | 106 (29) | 0.93 0.57-1.52) | 0.94 (0.57-1.55) | 54 (27) | 0.61 (0.29-1.27) | 0.61 (0.29-1.28) | 52 (32) | 1.37 (0.69-2.70) | 1.38 (0.69-2.78) |
|  | 4-7 days | 103 (29) | 0.95 (0.58-1.58) | 0.99 (0.59-1.64) | 55 (28) | 0.62 (0.30-1.29) | 0.62 (0.30-1.29) | 48 (30) | 1.41 (0.70-2.85) | 1.56 (0.75-3.22) |
| **LEGUMES** |  |  |  |  |  |  |  |  |  |  |
| Legumes e.g. baked beans, lentils | *Excessive* | 361 (48) |  |  | 199 (55) |  |  | 162 (42) |  |  |
|  | Never | 232 (67) | 1.00 (Ref) | 1.00 (Ref) | 127 (64) | 1.00 (Ref) | 1.00 (Ref) | 105 (33) | 1.00 (Ref) | 1.00 (Ref) |
|  | 1-3 days | 80 (22) | 0.92 (0.56-1.51) | 0.90 (0.55-1.48) | 47 (24) | 1.18 (0.55-2.51) | 1.19 (0.56-2.55) | 33 (20) | 0.73 (0.37-1.44) | 0.68 (0.34-1.36) |
|  | 4-7 days | 49 (14) | 1.08 (0.58-2.04) | 1.06 (0.56-2.01) | 25 (13) | 0.86 (0.36-2.08) | 0.91 (0.37-2.22) | 24 (15) | 1.37 (0.55-3.43) | 1.23 (0.47-3.23) |
|  |  |  |  |  |  |  |  |  |  |  |
| Peanut and nuts | *Excessive* | 361 (48) |  |  | 199 (55) |  |  | 162 (42) |  |  |
|  | Never | 286 (79) | 1.00 (Ref) | 1.00 (Ref) | 163 (82) | 1.00 (Ref) | 1.00 (Ref) | 123 (76) | 1.00 (Ref) | 1.00 (Ref) |
|  | 1-3 days | 45 (12) | 1.53 (0.74-3.15) | 1.51 (0.73-3.12) | 21 (11) | 1.26 (0.41-3.87) | 1.22 (0.39-3.81) | 24 (15) | 1.89 (0.73-4.87) | 1.92 (0.73-5.06) |
|  | 4-7 days | 30 (8) | 0.73 (0.37-1.43) | 0.74 (0.37-1.46) | 15 (8) | **0.33 (0.14-0.77)** | **0.34 (0.14-0.80)** | 15 (9) | 2.36 (0.66-8.48) | 2.48 (0.67-9.24) |
|  |  |  |  |  |  |  |  |  |  |  |
| **Fats/oils** | *Excessive* | 361 (48) |  |  | 199 (55) |  |  | 162 (42) |  |  |
| Soft margarine (tub) | Never | 112 (34) | 1.00 (Ref) | 1.00 (Ref) | 64 (32) | 1.00 (Ref) | 1.00 (Ref) | 48 (30) | 1.00 (Ref) | 1.00 (Ref) |
|  | 1-3 days | 87 (24) | 1.62 (0.92-2.87) | 1.59 (0.89-2.82) | 48 (24) | 1.57 (0.70-3.53) | 1.56 (0.68-3.60) | 39 (24) | 1.69 (0.75-3.80) | 1.68 (0.74-3.82) |
|  | 4-7 days | 162 (45) | 1.39 (0.87-2.21) | 1.41 (0.89-2.24) | 87 (44) | 1.56 (0.79-3.09) | 1.61 (0.82-3.18) | 75 (46) | 1.30 (0.68-2.48) | 1.33 (0.69-2.55) |

Adjusted model included maternal age, socio-economic status, relationship status, alcohol use and parity. Missing data n (%): Socio-economic status 2 (0.2), relationship status 5 (.05), alcohol use 1 (0.1). Where data are missing on predictors, cases were included in the reference category in the regression. Interpretation of OR’s: consumption of ‘food group’ for ‘frequency’ a week was associated with increased (OR>1) or decreases (OR<1) odds of excessive GWG compared to not consuming ‘food group’ in the past 7 days.
